# Supplementary material for: Impacts of ambient air quality on acute asthma hospital admissions during the COVID-19 pandemic in Oxford City, UK: a time-series study
Source: BMJ Open. 2024 Jan 22;14(1):e070704. doi: 10.1136/bmjopen-2022-070704 (PMC10806833; doi:10.1136/bmjopen-2022-070704)
Supplement: Supplementary data [file bmjopen-2022-070704supp001.pdf]

## *Supplementary material:*

# Impacts of ambient air quality on acute asthma hospital admissions during the COVID-19 pandemic in Oxford City, UK; a time series study

Ajit Singh [a, b, †], Gabriella L. Morley [c, †], Cécile Coignet [d], Felix Leach [e], Francis D. Pope [a], Neil Thomas [b], Brian Stacey [f], Tony Bush [e], Stuart Cole [g], George Economides [g], Ruth Anderson [g], Pedro Abreu [h] and Suzanne E. Bartington [b, *]

[a] School of Geography, Earth and Environmental Sciences, University of Birmingham, Edgbaston Park Road, Birmingham, B15 2TT, UK; a.singh.2@bham.ac.uk ; f.pope@bham.ac.uk

[b] Institute of Applied Health Research, University of Birmingham, Edgbaston Park Road, Birmingham, B15 2TT, UK; s.bartington@bham.ac.uk; g.n.thomas@bham.ac.uk

[c] Institute of Microbiology and Infection, University of Birmingham, Edgbaston Park Road, Birmingham, B15 2TT, UK; GLM092@student.bham.ac.uk

[d] NHS Oxfordshire Clinical Commissioning Group, Jubilee House, 5510 John Smith Drive, Oxford Business Park South, Oxford, OX4 2LH; cecile.coignet@nhs.nete

[e] Department of Engineering Science, University of Oxford, Parks Road, Oxford, OX1 3PJ, UK; felix.leach@eng.ox.ac.uk ; tony.bush@apertum.co.uk

[f] Ricardo Energy & Environment, Gemini Building, Fermi Avenue, Harwell, Oxfordshire, OX11 0QR. Email: brian.stacey@ricardo.com

[g] Oxfordshire County Council, County Hall, New Road, Oxford, OX1 1ND, UK; stuart.cole@oxfordshire.gov.uk; George.economides@oxfordshire.gov.uk; ruth.anderson@oxfordshire.gov.uk

[h] Oxford City Council, Town Hall, St Aldate's, Oxford, OX1 1BX; pabreu@oxford.gov.uk

†Joint first authors

*Correspondence to: s.bartington@bham.ac.uk (S.E.B)

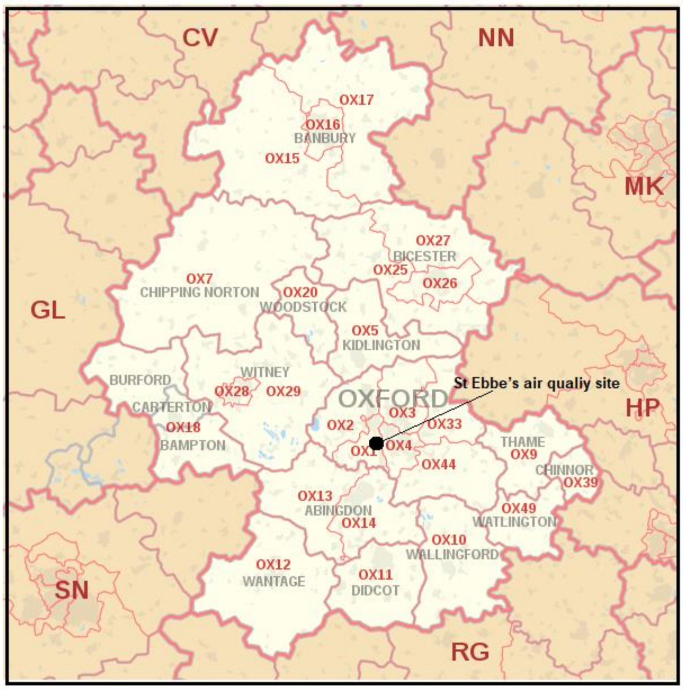


**Figure S1** Map of Oxford postcode districts illustrating Oxford City in OX1, OX2, OX3 and OX4. (Source, https://en.wikipedia.org/wiki/File:OX_postcode_area_map.svg).

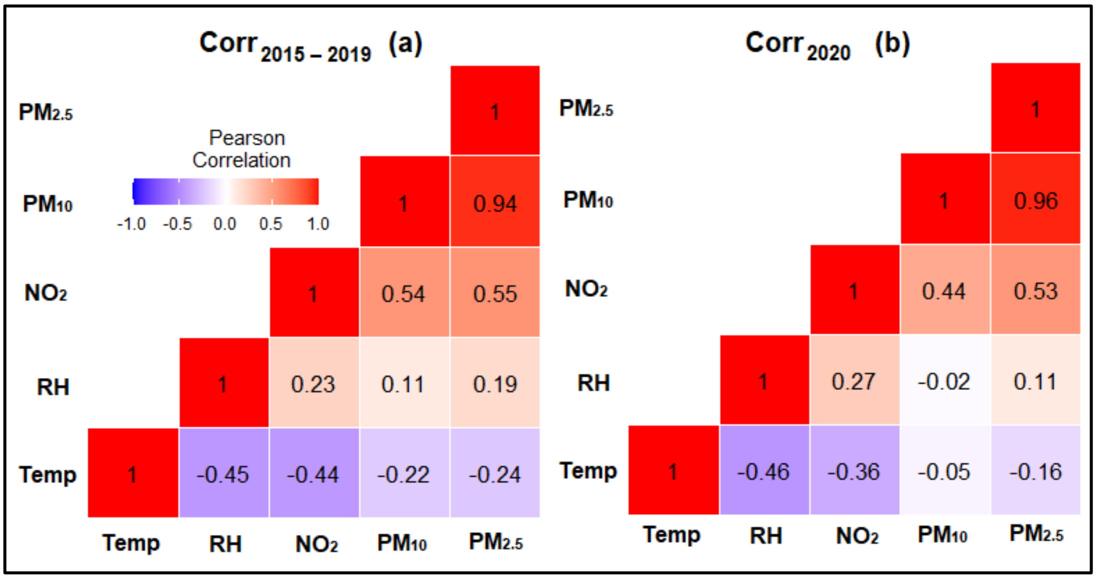


**Figure S2 Correlation** between daily mean air pollutant concentrations and meteorological variables during **a)** Covid-19 pandemic year (Corr*2020*) and **b)** pre-pandemic years (Corr*2015 – 2019*) in Oxford. Here, RH and Temp denote relative humidity and temperature, respectively.

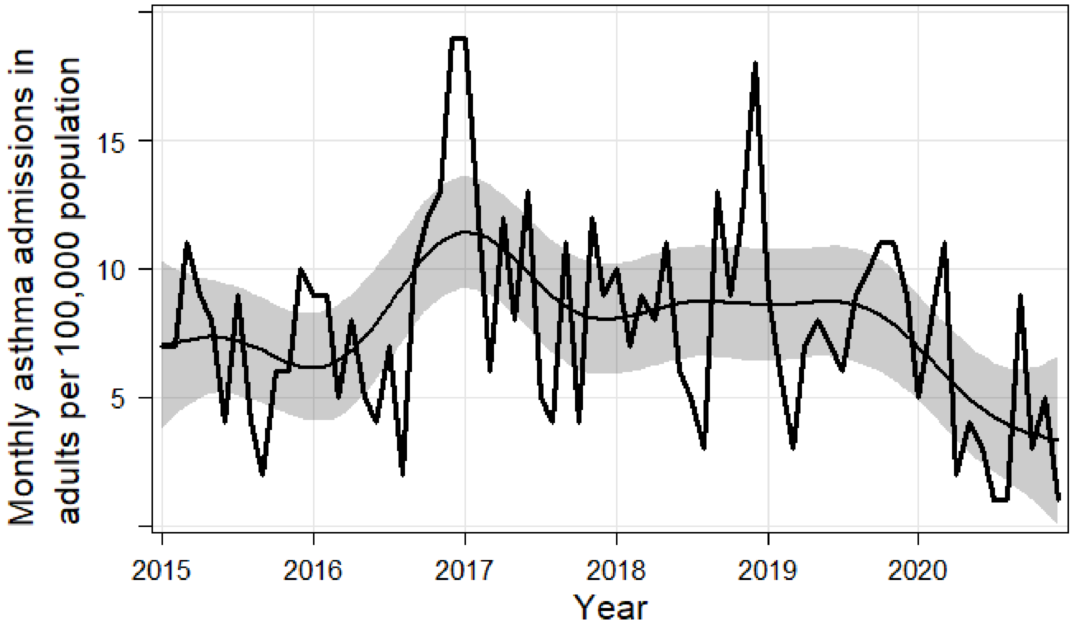


**Figure S3** Time series of monthly adult asthma admissions, where the shaded lines represent the smooth fit at the 95% confidence interval.

**Table S1** Incidence of adult hospital admissions for acute asthma in 2020 compared with pre-pandemic years (2015 – 2019).

| Period | Incidence rate | IRR | 95% CI | p value |
|---|---|---|---|---|
| **Pre-pandemic (2015 – 2019) mean** | 78.29 | Baseline | | |
| **2020** | 45.70 | 0.58 | 0.42 – 0.81 | <0.001 |

*per 100,000 population

**Table S2** Pearson correlation (r) matrix between air pollutant concentrations & meteorology and adult asthma admissions in 2020 versus 2015 – 2019**.**

| Variables | $NO_2$ | $PM_{2.5}$ | $PM_{10}$ | Temp | RH |
|---|---|---|---|---|---|
| **Asthma admission 2015 – 2019** | 0.31 | 0.27 | 0.22 | 0.34 | 0.30 |
| **Asthma admission 2020** | 0.22 | 0.16 | 0.20 | -0.38 | 0.14 |
