# Supplementary material for: Impacts of ambient air quality on acute asthma hospital admissions during the COVID-19 pandemic in Oxford City, UK: a time-series study
Source: BMJ Open. 2024 Jan 22;14(1):e070704. doi: 10.1136/bmjopen-2022-070704 (PMC10806833; doi:10.1136/bmjopen-2022-070704)
Supplement: Author's manuscript [file bmjopen-2022-070704.draft_revisions.pdf]

BMJ Open

BMJ Open is committed to open peer review. As part of this commitment we make the peer review history of every article we publish publicly available.

When an article is published we post the peer reviewers' comments and the authors' responses online. We also post the versions of the paper that were used during peer review. These are the versions that the peer review comments apply to.

The versions of the paper that follow are the versions that were submitted during the peer review process. They are not the versions of record or the final published versions. They should not be cited or distributed as the published version of this manuscript.

BMJ Open is an open access journal and the full, final, typeset and author-corrected version of record of the manuscript is available on our site with no access controls, subscription charges or pay-per-view fees (http://bmjopen.bmj.com).

If you have any questions on BMJ Open's open peer review process please email info.bmjopen@bmj.com

BMJ Open

Impacts of ambient air quality on acute asthma hospital admissions during the COVID-19 pandemic in Oxford City, UK; a time series study

Journal:	*BMJ Open*
Manuscript ID	bmjopen-2022-070704
Article Type:	Original research
Date Submitted by the Author:	09-Dec-2022

Complete List of Authors:	Singh, Ajit; University of Birmingham, School of Geography Earth and Environmental Sciences; University of Birmingham, Institute of Applied Health Research<br>Morley, Gabriella L.; University of Birmingham, Institute of Microbiology and Infection<br>Coignet, Cécile; NHS Oxfordshire CCG<br>Leach, Felix; University of Oxford, Department of Engineering Science<br>Pope, Francis; University of Birmingham, Geography Earth and Environmental Sciences<br>Neil Thomas, G.; University of Birmingham, Institute of Applied Health Research<br>Stacey, Brian; Ricardo-AEA Ltd<br>Bush, Tony; University of Oxford, Department of Engineering Science; Apertum<br>Cole, Stuart; Oxfordshire County Council<br>Economides, George; Oxfordshire County Council<br>Anderson, Ruth; Oxfordshire County Council<br>Abreu, Pedro; Oxford City Council<br>Bartington, Suzanne; University of Birmingham, Institute of Applied Health Research

Keywords:	Asthma < THORACIC MEDICINE, COVID-19, EPIDEMIOLOGIC STUDIES, Epidemiology < THORACIC MEDICINE, Health policy < HEALTH SERVICES ADMINISTRATION & MANAGEMENT

SCHOLARONE™
Manuscripts

I, the Submitting Author has the right to grant and does grant on behalf of all authors of the Work (as defined in the below author licence), an exclusive licence and/or a non-exclusive licence for contributions from authors who are: i) UK Crown employees; ii) where BMJ has agreed a CC-BY licence shall apply, and/or iii) in accordance with the terms applicable for US Federal Government officers or employees acting as part of their official duties; on a worldwide, perpetual, irrevocable, royalty-free basis to BMJ Publishing Group Ltd ("BMJ") its licensees and where the relevant Journal is co-owned by BMJ to the co-owners of the Journal, to publish the Work in this journal and any other BMJ products and to exploit all rights, as set out in our licence.

The Submitting Author accepts and understands that any supply made under these terms is made by BMJ to the Submitting Author unless you are acting as an employee on behalf of your employer or a postgraduate student of an affiliated institution which is paying any applicable article publishing charge ("APC") for Open Access articles. Where the Submitting Author wishes to make the Work available on an Open Access basis (and intends to pay the relevant APC), the terms of reuse of such Open Access shall be governed by a Creative Commons licence – details of these licences and which Creative Commons licence will apply to this Work are set out in our licence referred to above.

Other than as permitted in any relevant BMJ Author's Self Archiving Policies, I confirm this Work has not been accepted for publication elsewhere, is not being considered for publication elsewhere and does not duplicate material already published. I confirm all authors consent to publication of this Work and authorise the granting of this licence.

For peer review only

Impacts of ambient air quality on acute asthma hospital admissions during the COVID-19 pandemic in Oxford City, UK; a time series study

Ajit Singh [a, b, †], Gabriella L. Morley [c, †], Cécile Coignet [d], Felix Leach [e], Francis D. Pope [a], Neil Thomas [b], Brian Stacey [f], Tony Bush [e], Stuart Cole [g], George Economides [g], Ruth Anderson [g], Pedro Abreu [h] and Suzanne E. Bartington [b, *]

[a] School of Geography, Earth and Environmental Sciences, University of Birmingham, Edgbaston Park Road, Birmingham, B15 2TT, UK; a.singh.2@bham.ac.uk ; f.pope@bham.ac.uk

[b] Institute of Applied Health Research, University of Birmingham, Edgbaston Park Road, Birmingham, B15 2TT, UK; s.bartington@bham.ac.uk; g.n.thomas@bham.ac.uk

[c] Institute of Microbiology and Infection, University of Birmingham, Edgbaston Park Road, Birmingham, B15 2TT, UK; GLM092@student.bham.ac.uk

[d] NHS Oxfordshire Clinical Commissioning Group, Jubilee House, 5510 John Smith Drive, Oxford Business Park South, Oxford, OX4 2LH; cecile.coignet@nhs.nete

[e] Department of Engineering Science, University of Oxford, Parks Road, Oxford, OX1 3PJ, UK; felix.leach@eng.ox.ac.uk ; tony.bush@apertum.co.uk

[f] Ricardo Energy & Environment, Gemini Building, Fermi Avenue, Harwell, Oxfordshire, OX11 0QR. Email: brian.stacey@ricardo.com

[g] Oxfordshire County Council, County Hall, New Road, Oxford, OX1 1ND, UK; stuart.cole@oxfordshire.gov.uk; George.economides@oxfordshire.gov.uk; ruth.anderson@oxfordshire.gov.uk

[h] Oxford City Council, Town Hall, St Aldate's, Oxford, OX1 1BX; pabreu@oxford.gov.uk

†Joint first authors

*Correspondence to: s.bartington@bham.ac.uk (S.E.B)

For peer review only

For peer review only - http://bmjopen.bmj.com/site/about/guidelines.xhtml

Abstract

Objectives To investigate the short-term associations between exposure to ambient air pollution (NO_2, $PM_{2.5}$ and PM_{10}) and incidence of asthma hospital admissions among adults, in Oxford, UK.

Design Retrospective time-series study

Setting Oxford City (postcode areas OX1 – OX4), UK.

Participants Adult population living within the postcode areas OX1 - OX4 in Oxford, UK from 1 January 2015 to 31 December 2021

Primary and Secondary outcome measures Hourly NO_2, $PM_{2.5}$ and PM_{10} concentrations and meteorological data for the period 1 January 2015 to 31 December 2020 were analyzed and used as exposures. We used Poisson linear regression analysis to identify independent associations between air pollutant concentrations and asthma admissions rate among the adult study population, using both single (NO_2, $PM_{2.5}$, PM_{10}) and multi-pollutant (NO_2 and $PM_{2.5}$, NO_2 and PM_{10}) models, where they adjustment for temperature and relative humidity.

Results The overall five-year average asthma admissions rate was 78 per 100,000 population during the study period. The annual average rate decreased to 46 per 100,000 population during 2020 (IRR 0.58, 95% CI 0.42 to 0.81, p <0.001) compared to the pre-pandemic years (2015 – 2019). In single pollutant analysis we observed a significantly increased risk of asthma admission associated with each 1 μg m^{-3} increase in monthly concentrations of NO_2 4% (95% CI 1.009 to 1.072), $PM_{2.5}$ 3% (95% CI 1.006 – 1.052) and PM_{10} 1.8% (95% CI 0.999 – 1.038). However, in the multi-pollutant regression model, the effect of each individual pollutant was attenuated.

Conclusions Ambient NO_2 and $PM_{2.5}$ air pollution exposure increased the risk of asthma admissions in this urban setting. Improvements in air quality during COVID-19 lockdown periods may have contributed to a substantially reduced acute asthma disease burden. Large-scale measures to improve air quality have potential to protect vulnerable people living with chronic asthma in urban areas.

For peer review only - http://bmjopen.bmj.com/site/about/guidelines.xhtml

Article Summary

Strengths and limitations of this study

- This study investigated the association between ambient air pollution exposure and risk of admission to hospital for asthma
- The study includes an at-risk population of >800,000 person years
- Associations were evaluated using single and multi-pollutant models
- Causation cannot be inferred from this observational study
- A single city study may limits wider generalisation of findings

For peer review only

For peer review only - http://bmjopen.bmj.com/site/about/guidelines.xhtml

1. Introduction and background

Asthma is a non-communicable chronic disease characterized by airway hyper-responsiveness, reversible airflow obstruction and inflammation.[1] As the most common respiratory condition worldwide, asthma affects more than 300 million people globally including 12% of the population in the UK[2] with 4.3 million UK adults requiring treatment for the condition.[3] In 2019 asthma contributed to 368 Years Lived in Disability (YLD) (95% Confidence Interval (CI): 238–546) and 410 per 100,000 DALYs (95% CI: 280–588)[4] among the UK population, with the greatest disease burden occurring in areas of high deprivation.[3] Asthma can also be fatal and approximately 1200 people a year die from asthma in the UK.[5] As there is no permanent cure, clinical management is directed towards preventing or reducing the severity of exacerbations and controlling symptoms including wheeze, cough and breathlessness.[6 7] Primary prevention of asthma morbidity and mortality is therefore of major public health significance.

Asthma has multifactorial aetiology and is likely the result of complex gene and environmental interactions.[8] Environmental triggers may exacerbate symptoms so chronic asthma patients are advised to modify lifestyle and behaviours to reduce their exposure as a method for preventing exacerbations, alongside the use of inhaled or oral medication.[6] In recent years there has been increasing concern regarding the contribution of poor air quality to disease morbidity and mortality. Almost all of the world's population live in areas which do not meet 2021 WHO Global Air Quality Guidelines.[9-11] In the UK pollutants of major health concern include nitrogen dioxide (NO_2) which can cause airway inflammation, and fine particulate matter pollution – particles with diameter < 2.5 μm ($PM_{2.5}$), which can enter the circulation, with long term exposure to both pollutants linked to cardio-respiratory disease.[12-15] People with existing chronic respiratory diseases such as asthma or chronic obstructive pulmonary disease (COPD) are more vulnerable to $PM_{2.5}$ particle and NO_2 exposure[16] thereby exacerbating existing health inequalities. Long-term exposure to fine particulate matter ($PM_{2.5}$) and nitrogen dioxide (NO_2) is recognised to be causally associated with increased risk of developing child and adult asthma.[17 18] Mechanisms linking air pollution exposure to asthma include modification of inflammatory pathways, leading to airway remodeling and enhancement of sensitivity to environmental allergens.[19] A recent meta-analysis of 22 studies undertaken in 12 countries showed a significant

For peer review only - http://bmjopen.bmj.com/site/about/guidelines.xhtml

association between ambient air pollution exposure (NO_2, $PM_{2.5}$, CO and O_3) and risk of asthma exacerbations among adults and children. A five-year time series study (2009 – 2013) across primary care settings in London identified that an increase in short-term exposure to NO_2 and PM_{10} was associated with a significant increase in respiratory consultations, inhaler prescriptions, or both.[20]

Improvements in ambient air quality have also been linked to reduced asthma morbidity. Implementation of emergency public health measures in response to the COVID-19 pandemic, led to substantive reductions in industrial and transportation activities from spring 2020 and related changes in air pollution levels.[21-23] Studies in major cities worldwide indicated lockdown periods were associated with a reduction in major air pollutant (NO_2, PM_{10} and $PM_{2.5}$) concentrations.[24-29] Studies in the UK revealed a similar pattern to global trends in terms of air quality changes,[22 24 30] most notably NO_2 reductions in urban areas. [21]

The outbreak of COVID-19 led to concerns that this viral pandemic would increase the number of asthma exacerbations, resulting in an influx of emergency department visits and hospitalisations among asthma patients. However, a national analysis of anonymised patient records in Scotland and Wales identified substantial reductions in severe asthma exacerbations leading to hospital admission during COVID-19 lockdown periods, although mortality rates remained unchanged.[31] A reduction in attendance to primary care for asthma exacerbations was also identified in a large-scale analysis of routine data in England.[32] Similar reductions in asthma emergency attendances and admissions among children were also identified across 15 countries worldwide,[33] suggested to be due to reduced exposure triggers and increased treatment adherence. Whilst it is likely that the trends described in air pollution are replicated in smaller cities and towns, the majority of existing research has been conducted in major cities[24 30] with little opportunity to understand the relationship between changes in ambient air quality and asthma admissions among adults in smaller towns and cities in which 45% of the UK population live.[34]

In this study, a time-series analysis was conducted to investigate the association between ambient air pollution exposure and risk of unplanned (emergency) admissions among adults with asthma living in Oxford, UK.

For peer review only - http://bmjopen.bmj.com/site/about/guidelines.xhtml

2. Materials and methods

This study is part of the Natural Environment Research Council (NERC) funded OxAria Study' that aims to understand the impacts of COVID-19 on air and noise quality and associated health impacts in Oxford City (https://oxaria.org.uk/).

2.1 Study design

This study uses a retrospective time-series ecological design at the city level. The unit of analysis was a monthly count of the hospital admissions for adults, admitted with a primary diagnosis of acute asthma, during the study period of 1 January 2015 – 31 December 2020. Acute asthma is a serious condition involving progressive worsening of asthma symptoms, including breathlessness, wheeze, cough and chest tightness. Patients with severe of life-threatening acute asthma require immediate hospital treatment.

2.2 Setting – Oxford is a small historical city with an area of 46 km^2 and 68 m above sea level.[35] Oxford has a warm and temperate climate with mean annual temperature 10.3°C, mean annual cumulative rainfall 708 mm. The diverse population of this international city is approximately 152000, of which 79.6% are adults.[34] The city has two universities with approximately 34000 students enrolled. The city is a major centre for employment with a thriving economy, with an estimated 46000 people usually coming to the city for work, on a daily basis, before the COVID-19 pandemic.[36] The population is served by Oxford University Hospitals NHS Foundation Trust and comes under the jurisdiction of the Oxfordshire Clinical Commissioning Group (OCCG). A recent study by the OxAria team provides a detailed geographical, meteorological and environmental description of the study setting.[22]

2.3 Study population – The study population is adults aged >18 years living within Oxford City postcode areas OX1 – OX4 (see Figure 1).

2.4 Data collection

2.4.1 Health outcome data

Hospital Episode Statistics (HES) data was accessed through OCCG. The study sample were obtained from weekly aggregated HES admissions data to a single tertiary hospital (John Radcliffe Hospital, Oxford) where all patients admitted with a primary diagnosis of asthma, as the outcome of interest, were included. Cases were

For peer review only - http://bmjopen.bmj.com/site/about/guidelines.xhtml

extracted based on the inclusion criteria of age (>18 years), residential postcode district (OX1 – OX4) and Clinical Classifications identifier: ICD-10 (International Classification of Diseases 10_{th} Revision code) coding for acute asthma (ICD-10, J45 to J46).[37] Exclusion criteria were all cases of wheeze (ICD-10, R06.2), cough (ICD-10, R05) and influenza (ICD-10, J11). Missing values within the HES outcome dataset were minimal (4 %) over the total study duration. Missing values where present were replaced with the weekly average asthma cases for the study period. The mean monthly asthma hospital admissions rate was calculated and used as the unit of analysis.

2.4.2 Air pollution exposure and weather data

Hourly air pollutant concentrations (NO_2, $PM_{2.5}$ and PM_{10}) for the period 1 January 2015 to 31 December 2020 were obtained at the Urban and Rural Network (AURN) St Ebbe's urban background site (UKA00518),[38] located centrallyn in the OX1 – OX4 postcode area. Monthly mean air pollution concentrations were calculated from hourly datasets and analysed for the study period.

Hourly meteorological data including relative humidity (RH) and air temperature (T) from 1 January 2015 to 31 December 2020 were obtained from Oxford Radcliffe Observatory[39] – an urban background site, which was located in the OX2 postcode area The proportion of missing hourly data was 16% (NO_2), 20% (PM_{10}) and 24% ($PM_{2.5}$) across the six–year study duration. This was not replaced with imputed data given the potential for fluctuations over this time-period which may result in an inaccuracy or bias.

2.5 COVID-19 lockdown classification

Data were obtained from 1 January 2015 to 31 December 2020. We consider two national lockdown periods in England in 2020: (i) Lockdown 1 (23 March – 15 June 2020) and (ii) Lockdown 2 (5 November – 2 December 2020).[22 23]

2.6 Statistical analysis

Mean annual air pollutant concentrations (NO_2, $PM_{2.5}$, PM_{10}) for the study period (2015-2020) were calculated and compared to World Health Organisation Guideline levels.[11] Correlation between air pollutant concentrations and meteorological variables

For peer review only - http://bmjopen.bmj.com/site/about/guidelines.xhtml

(air temperature and relative humidity) was explored using Pearson correlation matrices and co-efficient.

The incidence rate of asthma admissions was determined for each year and the incidence rate ratio (IRR) compared to 2019 (baseline year), with a comparison of 2020 admissions rate to a five-year average by two-tailed t test. A Pearson correlation matrix was used to compare asthma admissions and air pollutant concentrations for 2020 and pre-pandemic years (2015 – 2019). To compare the trends between asthma admissions and air pollutant concentrations, values normalized by the mean were calculated and a time-series was generated.

Poisson linear regression analysis was used to quantify independent associations between monthly air pollutant concentrations and incidence of asthma admissions among the adult study population, using single (NO_2, $PM_{2.5}$, PM_{10}) and multi-pollutant (NO_2 and $PM_{2.5}$, NO_2 and PM_{10}) models, with adjustment for temperature and relative humidity. All statistical analysis was conducted in STATA 15 applying 95% confidence intervals and two-tailed p-values at <0.05 considered statistically significant.[40]

2.5 Patient and Public Involvement

The OxAria study was supported by involvement of members of the public who were consulted for the study duration and provided representation on the project advisory committee. In the early phase of the study a workshop was undertaken with members of Oxford Pedestrian's Association and Cyclox Oxford, who identified that asthma risk due to air pollution exposure was a particular priority concern among local residents. For this reason, asthma was selected as the health outcome of interest for further investigation. Study findings arising from air quality changes during COVID-19 lockdowsn have been made publicly available at https://oxaria.org.uk/ alongside being shared on social media channels and presented at a Friends of the Earth Oxford public webinar event. All findings will be made available in accessible formats, and hosted on the Oxfordshire Air Quality website, which is currently under development.

For peer review only

2. Results

2.1 Air quality

Overall mean annual average NO_2 and $PM_{2.5}$ concentrations for the total study duration exceeded the WHO Global Air Quality guideline average levels of 10 µg m^{-3} and 5 µg m^{-3}, for all study years respectively. A reduction in annual average NO_2, PM_{10} and $PM_{2.5}$ concentrations (0.74, 0.42 and 0.97 µg m^{-3} per year respectively) was observed from 2015 – 2019 which further reduced in 2020 (**Figure 2**). There was also evidence of seasonal peaks with higher pollutant levels in the winter months for all three pollutants (**Figure 2a**).

It is observed that the mean NO_2 concentration reduced by 26.7% to 10.7 µg m^{-3} in 2020 (95% CI 8.7 to 12.7 µg m^{-3}) which was significantly ($p<0.005$) lower than the five-year average (2015 – 2019) concentration of 14.6 µg m^{-3} (95% CI 12.6 to 16.7 µg m^{-3}). Similarly, the mean PM_{10} concentrations significantly ($p<0.05$) reduced by 18.6% in 2020 to 10.8 µg m^{-3} (95% CI 9.1 to 12.4 µg m^{-3}) compared with 13.2 µg m^{-3} (95% CI: 11.2 to 15.2 µg m^{-3}) from 2015 – 2019. Relatively, $PM_{2.5}$ levels showed a greater reduction than the other two pollutants, with mean $PM_{2.5}$ concentrations significantly ($p<0.005$) falling by 33.5% to 6.7 µg m^{-3} (95% CI 5.3 to 8.1 µg m^{-3}) in 2020 from 10.1 µg m^{-3} in 2015 – 2019 (95% CI 8.4 to 11.8 µg m^{-3}). However, a recent study using a machine learning for deweathering and detrending analysis has shown that these reductions were not only due to reduction in emissions but also influenced by the weather.[22]

$PM_{2.5}$ and PM_{10} levels were strongly positively correlated in both 2015-2019 (0.96) and 2020 (0.94) (**Figure 3**). A weakly positive correlation was also observed for NO_2 and $PM_{2.5}$, higher temperatures were associated with lower levels of air pollution, with a negative correlation between all temperature and all three pollutants although this was weaker in 2020. Relative humidity had a weakly positive association throughout the study period.

2.2 Asthma admissions

The overall incidence of asthma admissions for the total study period was 7.8 per month per 100,000 population. This varied by season, with the highest admissions rate during winter and spring, and the lowest in the summer months (**Figure 4 and 5**).

For peer review only - http://bmjopen.bmj.com/site/about/guidelines.xhtml

We found that there were two notable peaks in admissions of adults with asthma in late 2016 and 2019 that coincided with peaks in PM concentrations (see **Figure 2**). Overall, the asthma admissions rate in 2020 was 42% lower than the five-year average (IRR 0.58, p<0.001) (**Table 1**). There was no difference between the incidence of admissions in 2015 – 2018 compared with 2019, while there was a significantly reduced risk of admission in 2020 (IRR 0.6, p<0.01) compared to 2019 (**Table 2**).

Table 1 Incidence of adult hospital admissions for acute asthma in 2020 compared with pre-pandemic years (2015 – 2019).

Period	Incidence rate	IRR	95% CI	p value
**Pre-pandemic (2015 – 2019) mean**	78.29	Baseline		
**2020**	45.70	0.58	0.42 – 0.81	<0.001

*per 100,000 population

Table 2 Annual Incidence of adult hospital admissions for acute asthma by year (2015-2020), compared with 2019 as baseline.

Year	Annual Incidence rate	IRR	95% CI	p value
**2015**	62.78	0.83	(0.61 – 1.11)	p >0.05
**2016**	79.00	1.04	(0.79 – 1.38)	p >0.05
**2017**	91.59	1.20	(0.92 – 1.58)	p >0.05
**2018**	84.20	1.12	(0.84 – 1.46)	p >0.05
**2019**	76.08	Baseline		
**2020**	45.70	0.60	(0.43 – 0.82)	p <0.01

*per 100,000 population

For peer review only - http://bmjopen.bmj.com/site/about/guidelines.xhtml

Further, **Figure 5** shows the monthly count of hospital admissions of adults with asthma in 2020 and compares with the monthly average pre-epidemic years (2015 – 2019). We found that the number of admissions for the whole first pandemic year after March 2020 was lower than the five-year average. Although, there was a similar pattern in summer 2020 admissions compared to the five-year average summer, where the admission rate remained lower. In contrast, a higher peak was noted in March 2020 compared to the previous five years. We also observe a peak in admissions during September, however, again the admission rate remained below the five-year average. There was a marked decline in admissions during the preceding months in 2020 which was not seen during the pre-pandemic years.

2.3 Understating the effect of air quality on asthma admissions in adults in response to COVID-19 measures in Oxford

Monthly mean normalized time series of air pollutant (NO_2, PM_{10} and $PM_{2.5}$) concentrations and asthma admissions are presented in **Figure 6**. Overall, variation in asthma admissions among adults mostly followed trends in air pollution over the six-year study period, where a positive association between air pollutants and asthma admissions was observed in 2015 – 2019 with a relatively stronger correlation than in 2020 (**Figure 6 and Table 3**). However, there was a negative correlation between air temperature and asthma admissions in 2020 and whereas a positive correlation was observed in pre-pandemic years (**Table 3**).

Table 3 Pearson correlation (r) matrix between air pollutant concentrations & meteorology and adult asthma admissions in 2020 versus 2015 – 2019.

Variables	NO_2	$PM_{2.5}$	PM_{10}	Temp	RH
**Asthma admission 2015 – 2019**	0.31	0.27	0.22	0.34	0.30
**Asthma admission 2020**	0.22	0.16	0.20	-0.38	0.14

Further, to understand the association between air pollutants and asthma admissions among adults, single-pollutant and multi-pollutant models were generated applying Poisson regression analysis (**Table 4**). In the single-pollutant model, which was adjusted for temperature and relative humidity during the study period, showed that the risk of asthma admission increased significantly with increasing concentrations of

For peer review only - http://bmjopen.bmj.com/site/about/guidelines.xhtml

NO_2 and $PM_{2.5}$. This was a 4% increase in risk for every 1 μg m^{-3} increase in mean monthly NO_2 and an approximately 3% increase in risk for every 1 μg m^{-3} increase in mean monthly $PM_{2.5}$. For every 1 μg m^{-3} increase in mean monthly PM_{10}, there was also a 1.8% increase in the risk of asthma admission, however, this failed to reach significance despite narrow confidence intervals.

Within the multi-pollutant model, the effect of each individual pollutant was attenuated. A weak correlation between pollutants makes it difficult for the model to separate the effects of correlated pollutants. Overall, in the multi-pollutants model we identified a non-significant 2.6% increase in risk of asthma admission per month predicted for every 1 μg m^{-3} increase in NO_2 and a non-significant 1.7% increase in risk of admission for every 1 μg m^{-3} increase $PM_{2.5}$, which approximates the risk for PM_{10}.

Table 4 Incidence rate ratio (IRR) for adult asthma admissions of in response to changes in air pollutant concentrations using the Poisson model.

Pollutants	Single-pollutant model			Multi-pollutant model		
	IRR	95% CI	p value	IRR	95% CI	p value
NO_2	1.040	1.009 – 1.072	$p < 0.05$	1.026	0.987 – 1.067	$p > 0.05$
$PM_{2.5}$	1.029	1.006 – 1.052	$p < 0.05$	1.017	0.988 – 1.045	$p > 0.05$
PM_{10}	1.018	0.999 – 1.038	$p > 0.05$	NA	NA	NA

3. Discussion

This study examines for the first time the effects of ambient air quality on asthma related hospital admissions in adults in Oxford city (UK), including impacts of the COVID-19 pandemic. A positive association was observed between risk of emergency asthma admission and air pollutant levels in Oxford, with hospital admission risk significantly increased even with modest increases in mean monthly NO_2 and $PM_{2.5}$ concentrations in single pollutant models. We also found a 42% reduction in the risk of acute asthma admission among adults living in Oxford in 2020 compared to the five-year average, suggesting air quality improvements during COVID-19 lockdown

For peer review only - http://bmjopen.bmj.com/site/about/guidelines.xhtml

periods may have contributed to a reduction in severe asthma exacerbations. However, this observed association is in the context of a general reduction in emergency admissions for all causes during the COVID-19 period.

The findings of this work, supported by recent studies, found that pollution levels – notably NO_2 concentrations - decreased significantly in 2020 compared to pre-pandemic years[22 23 41 42] due to changes in anthropogenic activities This pattern needs to considered in the context of already declining levels of air pollution in Oxford and exceptional weather in 2020, including a warmer and windier early part of the year, coinciding with the first national lockdown. [22 41] Recent studies by the OxAria team highlighted a clear reduction in traffic volume of 69% and 38% during the first and second lockdowns, respectively in Oxford, where NO_2 emissions from buses and cars reduced by 56% and 77% respectively.[22 23] Whilst we did not attempt to explore contributions of specific emissions sources to changes in asthma admissions, the statistically significant effect of NO_2 and $PM_{2.5}$ in the single-pollutant model was not replicated in the multi-pollutant model, which indicates that changes in emissions sources may be relevant for determining overall asthma exacerbation risk in this context.

The findings presented in our study regarding the consistently positive association between NO_2 and $PM_{2.5}$ and asthma exacerbations rates are consistent with the existing evidence.[43-52] The identified lockdown impact is also broadly consistent with a large-scale analysis of emergency admissions in Scotland and Wales which showed a 36% reduction in asthma related hospitalizations for all-ages population during the first 18 weeks of 2020,[31] however the fixed-effect meta-analysis model limited wider generalisability. Davies and colleagues proposed multiple possible theories for their observations, including reductions in NO_2 and $PM_{2.5}$ that were a national trend, however, unfortunately they were unable to substantiate this proposition as they did not collect concurrent air quality data. Further there exist differences in healthcare access and COVID-19 emergency public health regulation between the devolved nations and England. The first pandemic year, 2020 was also exceptional for the low rates of asthma admissions worldwide,[53] as previously reported in the paediatric population[54-58] and reflected in our own local admissions dataset.

For peer review only - http://bmjopen.bmj.com/site/about/guidelines.xhtml

There are other hypotheses for the most likely contributing factors to the reduction in asthma admissions in 2020, including speculation that patients were more vigilant with their asthma prescriptions and therefore had improved compliance to preventative inhaled steroid medication.[31] Given the fear about contracting COVID-19 when also diagnosed with a chronic condition [59] and the findings from the RECOVERY trial with adoption of steroids as a treatment for COVID-19,[60] it is plausible that patients improved compliance with their asthma plan, including compliance with any corticosteroid medication, during the pandemic and this contributed to a reduction in admissions during 2020.[31] This explanation has not been widely tested however, and it is unlikely that compliance continued throughout the entirety of 2020. An alternative theory is that many patients avoided healthcare services either due to fear that they may contract COVID-19 or a moral sense of duty to prevent overwhelming the NHS.[59 61 62] There is weak evidence though for patients with asthma delaying presentation given there was no change in asthma mortality and no increase in acuity of admissions from the studies which looked at this metric.[63 64] This suggests that delays in seeking healthcare assistance was not a reason for the lower admission rates observed here. Furthermore, asthma admission was chosen instead of attendance for the outcome in this study to reduce the chance that the change observed was due to a change in patient behaviour (delay in presentation). Nevertheless, it would have been beneficial for this study and any future studies to include a mechanism for measuring the extent to which a patient delayed presentation. Some studies have suggested that the low asthma admission rate may be due to low person-to-person transmission of other respiratory viruses, however this is unlikely to completely explain the dramatic reduction in 2020 admissions observed in multiple countries as well as the UK.[31 61 65 66]

Strengths and limitations – This study has some additional limitations including the ecological, observational design that precludes the establishment of causality. Due to the observational study design it is difficult to conclude that changes in air pollution concentrations were wholly responsible for the reduction in asthma admissions during 2020. During the early pandemic phase several reports suggested that all-cause emergency admissions reduced, as the public responded to the “protect the NHS” campaign or were too fearful of coronavirus risks to present to hospital.[61] However, acute asthma is a severe condition which requires urgent hospital management and

For peer review only - http://bmjopen.bmj.com/site/about/guidelines.xhtml

therefore it is likely that most patients in this study accessed emergency care following an acute attack. Our findings are also consistent with those of Shah and colleagues, who identified a statistically significant reduction in asthma patients who experienced an exacerbation during the lockdown period. The aggregated nature of the data collected on asthma admissions and the estimates of the population at risk means that caution must be exercised regarding any inferences at the individual level; despite a defined population at risk, within a relatively small pollutant area, it is not known whether those admitted with asthma were exposed to the air pollutant concentrations measured. Finally, we include data for ambient pollutant exposure only and recognise that adult patients with diagnosed asthma are likely to have spent a high duration of time indoors, particularly during lockdown periods. Further research is required to understand the respective changes in indoor and ambient pollutant concentrations during the COVID-19 pandemic and to better understand links to chronic disease outcomes among vulnerable patients. In particular, further studies should analyse admissions at the patient-level to understand if pollutants have a particular impact on specific subgroups of the population with asthma which could be used to target future public health policies and guidance.

4. Conclusions

This study provides important insights into effects of changes in ambient air quality on asthma exacerbations in adults during the COVID-19 pandemic in Oxford, UK. The COVID-19 national lockdown measures have led to pronounced changes in air pollutant (NO_2, PM_{10} and $PM_{2.5}$) concentrations in Oxford. Our results show that adults with asthma had significantly lower risk of unplanned hospital admission in 2020 as compared to the pre-pandemic years (2015 – 2019). While this study cannot attribute exact causation, our findings suggest that improvements to air quality could reduce asthma healthcare service burden. There is a need to identify which pollutants, sources and locations would be most beneficial to target with future public health interventions to prevent severe asthma exacerbations requiring hospitalization in adults.

For peer review only - http://bmjopen.bmj.com/site/about/guidelines.xhtml

Author Contributions: S.E.B, F.L, F.D.P, G.N.T, B.S, T.B, S.C, G.E contributed to funding acquisition for the wider OxAria study. S.E.B, G.M and G.N.T conceived and designed the current study. R.A and C.C. acquired linked health data. T.B and P.A acquired air quality and meteorological data. G.M linked air quality and health data, conducted initial data analysis and drafted the initial report under the supervision of S.E.B. A.S undertook data analysis, wrote the draft article, and undertook data visualisation. All authors contributed to editing and critically revising the article and approved the final draft. S.E.B is guarantor of the work.

Acknowledgments We are grateful to staff at Oxfordshire Clinical Commissioning Group, Oxfordshire County Council and Oxford City Council for supporting this work. We wish to thank all members of the OxAria Study Advisory Group, including Chair Professor Neil Harris and former Chair Professor Martin Williams for providing advice and feedback. We are grateful to members of Oxford Pedestrian's Association for providing input to the design and conduct of this study, notably Ms Sushila Dhal and Keith Frayn who provided lay member representation for the OxAria Advisory Group.

Funding This research was funded by the Natural Environment Research Council (grant no. NE/V010360/1). Its forerunning pilot project was funded by the National Institute for Health and Care Research (NIHR130095; NIHR Public Health Research). The views expressed are those of the author(s) and not necessarily those of the NIHR or the Department of Health and Social Care.

Data Availability Statement Air quality data is available from https://uk-air.defra.gov.uk/data/data_selector.

Conflicts of Interest: The authors declare no conflict of interest.

Ethical statement Ethical approval for the OxAria study was provided by the University of Birmingham Research Ethics Committee (ERN_20-0831).

For peer review only

References

1. Holgate ST, Thomas M. Chapter 7 - Asthma. In: O'Hehir RE, Holgate ST, Sheikh A, eds. Middleton's Allergy Essentials: Elsevier 2017:151-204.
2. NICE. What is the prevalence of asthma? National Institute for Health and Care Excellence, London, United Kingdom,2022. Available: https://cks.nice.org.uk/topics/asthma/background-information/prevalence/ [Accessed 15 May 2022]
3. ALU. Asthma UK, Asthma facts and statistics. Asthma + Lung UK, London, United Kingdom, 2022. Available: https://www.asthmaandlung.org.uk/media-centre/ [Accessed 10 April 2022]
4. IHME. The global burden of disease study, Institute for Health Metrics and Evaluation, University of Washington, USA, 2022. Available: https://vizhub.healthdata.org/gbd-compare/ [Accessed 28 September 2022]
5. BLF. Asthma statistics. British Lung Foundation, London, United Kingdom, 2022. Available: https://statistics.blf.org.uk/asthma [Accessed 15 May 2022]
6. NICE. Asthma: diagnosis, monitoring and chronic asthma management. National Institute for Health and Care Excellence, London, United Kingdom, 2021. Available: https://www.nice.org.uk/guidance/ng80/chapter/Recommendations [Accessed 15 May 2022]
7. NICE. Scenario: Newly-diagnosed asthma. National Institute for Health and Care Excellence, London, United Kingdom, 2021. https://cks.nice.org.uk/topics/asthma/management/newly-diagnosed-asthma/ [Accessed 15 May 2022]
8. Subbarao P, Mandhane PJ, Sears MR. Asthma: epidemiology, etiology and risk factors. *Canadian Medical Association Journal* 2009;**181**(9):E181. doi:10.1503/cmaj.080612
9. WHO. WHO's Urban Ambient Air Pollution Database–Update 2016. Geneva: World Health Organization, 2016. Available: https://cdn.who.int/media/docs/default-source/air-quality-database/aqd-2016/aap_database_summary_results_2016_v02.pdf?sfvrsn=384beb23_3 [Accessed 15 May 2022]
10. WHO. Billions of people still breathe unhealthy air: new WHO data. World Health Organization, Geneva, Switzerland, 2022. Available: https://www.who.int/news/item/04-04-2022-billions-of-people-still-breathe-unhealthy-air-new-who-data [Accessed 15 May 2022]
11. WHO. WHO global air quality guidelines: particulate matter ($PM_{2.5}$ and PM_{10}), ozone, nitrogen dioxide, sulfur dioxide and carbon monoxide. World Health Organization, Geneva, Switzerland, 2021. Available: https://apps.who.int/iris/handle/10665/345329 [Accessed 15 May 2022]
12. Madureira J, Brancher EA, Costa C, et al. Cardio-respiratory health effects of exposure to traffic-related air pollutants while exercising outdoors: A systematic review. *Environmental Research* 2019;**178**:108647. doi:10.1016/j.envres.2019.108647
13. PHE. Health matters: air pollution. Public Health England, London, United Kingdom, 2018. Available: https://www.gov.uk/government/publications/health-matters-air-pollution/health-matters-air-pollution [Accessed 15 May 2022]
14. PHE. Review of interventions to improve outdoor air quality and public health. Public Health England, London, United Kingdom, 2019. Available:

For peer review only - http://bmjopen.bmj.com/site/about/guidelines.xhtml

https://assets.publishing.service.gov.uk/government/uploads/system/uploads/attachment_data/file/938623/Review_of_interventions_to_improve_air_quality_March-2019-2018572.pdf [Accessed 15 May 2022]

15. ONS. Road transport and air emissions. Office for National Statistics, London, United Kingdom, 2019. https://www.ons.gov.uk/economy/environmentalaccounts/articles/roadtransportandairemissions/2019-09-16 [Accessed 15 May 2022]

16. Jiang X-Q, Mei X-D, Feng D. Air pollution and chronic airway diseases: what should people know and do? *Journal of Thoracic Disease* 2015;**8**(1):E31-E40. doi:10.3978/j.issn.2072-1439.2015.11.50

17. Jacquemin B, Siroux V, Sanchez M, et al. Ambient Air Pollution and Adult Asthma Incidence in Six European Cohorts (ESCAPE). *Environmental Health Perspectives* 2015;**123**(6):613-21. doi:10.1289/ehp.1408206

18. Khreis H, Kelly C, Tate J, et al. Exposure to traffic-related air pollution and risk of development of childhood asthma: A systematic review and meta-analysis. *Environment International* 2017;**100**:1-31. doi:10.1016/j.envint.2016.11.012

19. COMEAP. Committee on the Medical Effects of Air Pollutants. Centre for Radiation, Chemical and Environmental Hazards, Chilton, United Kingdom, 2022. Available: https://www.gov.uk/government/groups/committee-on-the-medical-effects-of-air-pollutants-comeap [Accessed 15 May 2022]

20. Ashworth M, Analitis A, Whitney D, et al. Spatio-temporal associations of air pollutant concentrations, GP respiratory consultations and respiratory inhaler prescriptions: a 5-year study of primary care in the borough of Lambeth, South London. *Environmental Health* 2021;**20**(1):54. doi:10.1186/s12940-021-00730-1

21. AQEG. Report: Estimation of changes in air pollution emissions, concentrations and exposure during the COVID-19 outbreak in the UK: Rapid evidence review. The Air Quality Expert Group, Department for Environment, Food and Rural Affairs (Defra), London, United Kingdom, 2020. Available: https://uk-air.defra.gov.uk/assets/documents/reports/cat09/2007010844_Estimation_of_Changes_in_Air_Pollution_During_COVID-19_outbreak_in_the_UK.pdf [Accessed 15 May 2022]

22. Singh A, Bartington SE, Song C, et al. Impacts of emergency health protection measures upon air quality, traffic and public health: evidence from Oxford, UK. *Environmental Pollution* 2022;**293**:118584. doi:10.1016/j.envpol.2021.118584

23. Singh A, Guo T, Bush T, et al. Impacts of COVID-19 lockdown on traffic flow, active travel and gaseous pollutant concentrations; implications for future emissions control measures in Oxford, UK. Research Square [Preprint]. April 19, 2022 [cited 2022 May 15] https://doi.org/10.21203/rs.3.rs-1490381/v1

24. Shi Z, Song C, Liu B, et al. Abrupt but smaller than expected changes in surface air quality attributable to COVID-19 lockdowns. *Science Advances* 2021;**7**(3):eabd6696. doi:10.1126/sciadv.abd6696

25. Mahato S, Pal S, Ghosh KG. Effect of lockdown amid COVID-19 pandemic on air quality of the megacity Delhi, India. *Science of the Total Environment* 2020;**730**:139086. doi:10.1016/j.scitotenv.2020.139086

26. Kumar P, Hama S, Omidvarborna H, et al. Temporary reduction in fine particulate matter due to 'anthropogenic emissions switch-off'during COVID-19 lockdown in Indian cities. *Sustainable cities and society* 2020;**62**:102382.

For peer review only - http://bmjopen.bmj.com/site/about/guidelines.xhtml

27. Baldasano JM. COVID-19 lockdown effects on air quality by NO2 in the cities of Barcelona and Madrid (Spain). *Science of the Total Environment* 2020;**741**:140353. doi:10.1016/j.scs.2020.102382
28. Lian X, Huang J, Huang R, et al. Impact of city lockdown on the air quality of COVID-19-hit of Wuhan city. *Science of the Total Environment* 2020;**742**:140556. doi:10.1016/j.scitotenv.2020.140556
29. Liu F, Wang M, Zheng M. Effects of COVID-19 lockdown on global air quality and health. *Science of The Total Environment* 2021;**755**:142533. doi:10.1016/j.scitotenv.2020.142533
30. Jephcote C, Hansell AL, Adams K, et al. Changes in air quality during COVID-19 'lockdown' in the United Kingdom. *Environmental Pollution* 2021;**272**:116011. doi:10.1016/j.envpol.2020.116011
31. Davies GA, Alsallakh MA, Sivakumaran S, et al. Impact of COVID-19 lockdown on emergency asthma admissions and deaths: national interrupted time series analyses for Scotland and Wales. *Thorax* 2021;**76**(9):867. doi: 10.1136/thoraxjnl-2020-216380
32. Shah SA, Quint JK, Nwaru BI, et al. Impact of COVID-19 national lockdown on asthma exacerbations: interrupted time-series analysis of English primary care data. *Thorax* 2021;**76**(9):860. doi:10.1136/thoraxjnl-2020-216512
33. Papadopoulos NG, Mathioudakis AG, Custovic A, et al. Childhood asthma outcomes during the COVID-19 pandemic: Findings from the PeARL multi-national cohort. *Allergy* 2021;**76**(6):1765-75. doi:10.1111/all.14787
34. ONS. Population estimates for the UK, England and Wales, Scotland and Northern Ireland: mid-2019, using April 2019 local authority district codes. Office for National Statistics, Fareham, England, 2020. Available: https://www.ons.gov.uk/peoplepopulationandcommunity/populationandmigration/populationestimates/datasets/populationestimatesforukenglandandwalesscotlandandnorthernireland [Accessed 18 May 2021]
35. OP AM. AM Online Projects. Oxford, United Kingdom, 2022. Available: https://en.climate-data.org/europe/united-kingdom/england/oxford-22/ [Accessed 18 May 2022]
36. OCC. Oxford Profile 2018 Key Facts Oxford City Council. Oxford, United Kingdom, 2018. Available: https://www.oxford.gov.uk/downloads/file/5021/oxford_profile_2018 [Accessed 18 May 2022]
37. ICD. International Statistical Classification of Diseases and Related Health Problems 10th Revision (ICD-10) Version for 2010. International Classification of Diseases, United Nations System, 2010. Available: https://icd.who.int/browse10/2010/en [Accessed 18 May 2022]
38. DEFRA. UK air information. Department for Environment Food & Rural Affairs, United Kingdom, 2022. Available: https://uk-air.defra.gov.uk [Accessed 10 April 2022]
39. SoGE. Daily data from the Radcliffe Observatory site in Oxford. School of Geography and Environment, University of Oxford, Oxford, United Kingdom, 2022. Available: https://www.geog.ox.ac.uk/research/climate/rms/daily-data.html [Accessed 10 April 2022)
40. STATA. Stata 15: Statistical software for data science, 2022. Available: https://www.stata.com/stata15/ [Accessed 10 April 2022]

For peer review only - http://bmjopen.bmj.com/site/about/guidelines.xhtml

41. Carslaw D. Blog update on COVID-19 and changes in air pollution. Ricardo, United Kingdom. Available: https://ee.ricardo.com/news/blog-update-on-covid-19-and-changes-in-air-pollution [Accessed 18 May 2022]
42. Abreu P. Air Quality Annual Status Report. Oxford City Council, Oxford, United Kingdom, 2021. Available: https://www.oxford.gov.uk/downloads/file/7612/air_quality_annual_status_report_2020 [Accessed 18 May 2022]
43. Orellano P, Quaranta N, Reynoso J, et al. Effect of outdoor air pollution on asthma exacerbations in children and adults: systematic review and multilevel meta-analysis. *PloS one* 2017;**12**(3):e0174050. doi:10.1371/journal.pone.0174050
44. Guarnieri M, Balmes JR. Outdoor air pollution and asthma. *The Lancet* 2014;**383**(9928):1581-92. doi:10.1016/S0140-6736(14)60617-6
45. Sunyer J, Spix C, Quénel P, et al. Urban air pollution and emergency admissions for asthma in four European cities: the APHEA Project. *Thorax* 1997;**52**(9):760. doi:10.1136/thx.52.9.760
46. Zhang Y, Ni H, Bai L, et al. The short-term association between air pollution and childhood asthma hospital admissions in urban areas of Hefei City in China: A time-series study. *Environmental Research* 2019;**169**:510-16. doi:10.1016/j.envres.2018.11.043
47. Castner J, Guo L, Yin Y. Ambient air pollution and emergency department visits for asthma in Erie County, New York 2007–2012. *International Archives of Occupational and Environmental Health* 2018;**91**(2):205-14. doi:10.1007/s00420-017-1270-7
48. Galán I, Tobías A, Banegas JR, et al. Short-term effects of air pollution on daily asthma emergency room admissions. *European Respiratory Journal* 2003;**22**(5):802. doi:10.1183/09031936.03.00013003
49. Jacquemin B, Schikowski T, Carsin AE, et al. The Role of Air Pollution in Adult-Onset Asthma: A Review of the Current Evidence. *Seminars in Respiratory and Critical Care Medicine* 2012;**33**(06):606-19. doi:10.1055/s-0032-1325191
50. Tiotiu AI, Novakova P, Nedeva D, et al. Impact of Air Pollution on Asthma Outcomes. *International Journal of Environmental Research and Public Health* 2020; **17**(17): 6212. doi:10.3390/ijerph17176212
51. Kuo C-Y, Chan C-K, Wu C-Y, et al. The Short-Term Effects of Ambient Air Pollutants on Childhood Asthma Hospitalization in Taiwan: A National Study. *International Journal of Environmental Research and Public Health* 2019;**16**(2):203. doi:10.3390/ijerph16020203
52. Lam HC-y, Li AM, Chan EY-y, et al. The short-term association between asthma hospitalisations, ambient temperature, other meteorological factors and air pollutants in Hong Kong: a time-series study. *Thorax* 2016;**71**(12):1097. doi:10.1136/thoraxjnl-2015-208054
53. Onay ZR, Mavi D, Ayhan Y, et al. Did Hospital Admissions Caused by Respiratory Infections and Asthma Decrease During the COVID-19 Pandemic? *Medeniyet Medical Journal* 2022;**37**(1):92-98. doi:10.4274/MMJ.galenos.2022.02779
54. Krivec U, Kofol Seliger A, Tursic J. COVID-19 lockdown dropped the rate of paediatric asthma admissions. *Archives of Disease in Childhood* 2020;**105**(8):809. doi:10.1136/archdischild-2020-319522
55. Williams TC, MacRae C, Swann OV, et al. Indirect effects of the COVID-19 pandemic on paediatric healthcare use and severe disease: a retrospective

national cohort study. *Archives of Disease in Childhood* 2021;**106**(9):911. doi:10.1136/archdischild-2020-321008
56. Dann L, Fitzsimons J, Gorman KM, et al. Disappearing act: COVID-19 and paediatric emergency department attendances. *Archives of Disease in Childhood* 2020;**105**(8):810. doi:10.1136/archdischild-2020-319654
57. Ashikkali L, Carroll W, Johnson C. The indirect impact of COVID-19 on child health. *Paediatrics and Child Health* 2020;**30**(12):430-37. doi:10.1016/j.paed.2020.09.004
58. Vásquez-Hoyos P, Diaz-Rubio F, Monteverde-Fernandez N, et al. Reduced PICU respiratory admissions during COVID-19. *Archives of Disease in Childhood* 2021;**106**(8):808. doi:10.1136/archdischild-2020-320469
59. Lazzerini M, Barbi E, Apicella A, et al. Delayed access or provision of care in Italy resulting from fear of COVID-19. *The Lancet Child & Adolescent Health* 2020;**4**(5):e10-e11. doi:10.1016/S2352-4642(20)30108-5
60. Group TWREAfC-TW. Association Between Administration of Systemic Corticosteroids and Mortality Among Critically Ill Patients With COVID-19: A Meta-analysis. *JAMA* 2020;**324**(13):1330-41. doi:10.1001/jama.2020.17023
61. Mulholland RH, Wood R, Stagg HR, et al. Impact of COVID-19 on accident and emergency attendances and emergency and planned hospital admissions in Scotland: an interrupted time-series analysis. *Journal of the Royal Society of Medicine* 2020;**113**(11):444-53. doi:10.1177/0141076820962447
62. TFLH. Over a third of people with lung conditions felt pressure to avoid or delay seeking treatment during lockdown London. Taskforce for Lung Health, London, United Kingdom, 2020. Available: https://www.blf.org.uk/taskforce/press-release/over-a-third-of-people-with-lung-conditions-felt-pressure-to-avoid-or-delay-seeking-treatment [Accessed 09 July 2022].
63. Chavasse R, Almario A, Christopher A, et al. The Indirect Impact of COVID-19 on Children With Asthma. *Archivos de Bronconeumología* 2020;**56**(11):768-69. doi:10.1016/j.arbres.2020.07.003
64. Sykes DL, Faruqi S, Holdsworth L, et al. Impact of COVID-19 on COPD and asthma admissions, and the pandemic from a patient's perspective. *ERJ Open Research* 2021;**7**(1):00822-2020. doi:10.1183/23120541.00822-2020
65. Abe K, Miyawaki A, Nakamura M, et al. Trends in hospitalizations for asthma during the COVID-19 outbreak in Japan. *The Journal of Allergy and Clinical Immunology: In Practice* 2021;**9(**1):494-96.e1. doi:10.1016/j.jaip.2020.09.060
66. Jones N. How coronavirus lockdowns stopped flu in its tracks. Nature news. Available: *https://wwwnaturecom/articles/d41586-020-01538-8* [Accessed 09 July 2022].

For peer review only - http://bmjopen.bmj.com/site/about/guidelines.xhtml

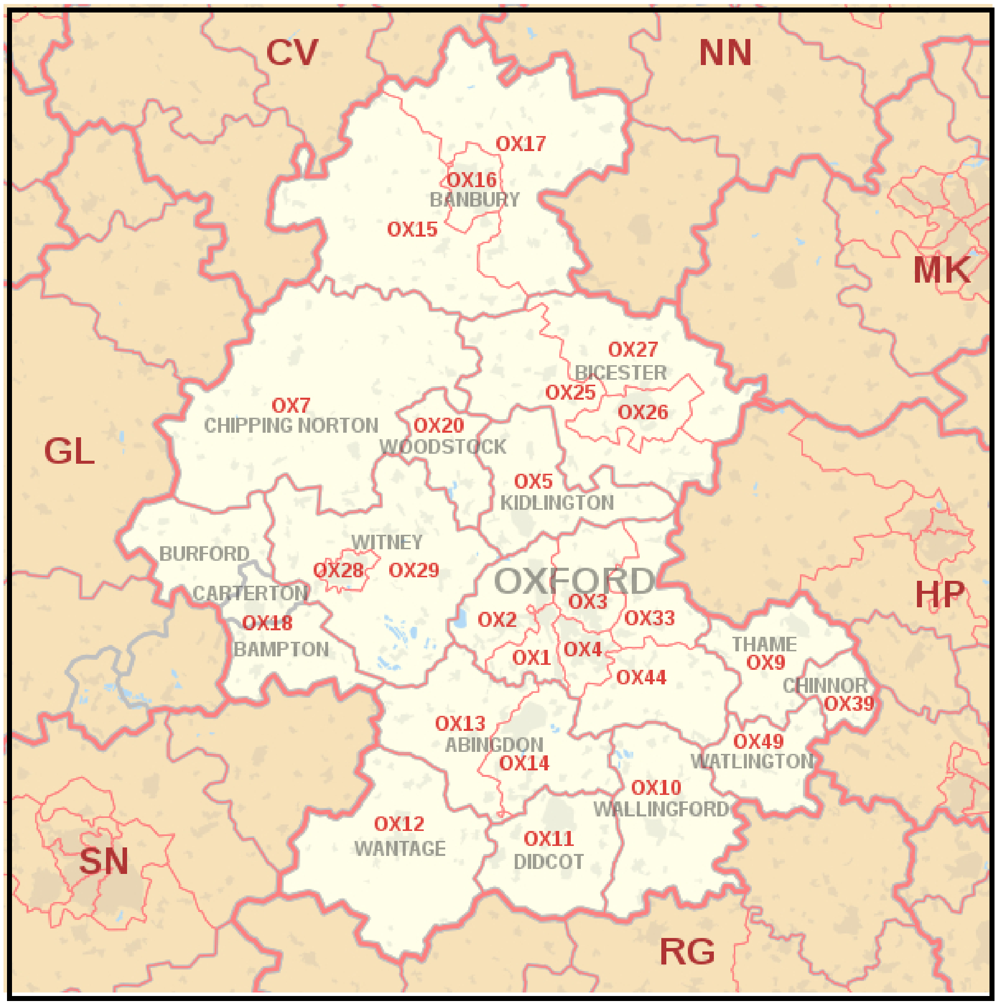

Figure 1. Map of Oxford postcode districts illustrating Oxford City in OX1, OX2, OX3 and OX4. (Source: https://en.wikipedia.org/wiki/File:OX_postcode_area_map.svg).

405x411mm (38 x 38 DPI)

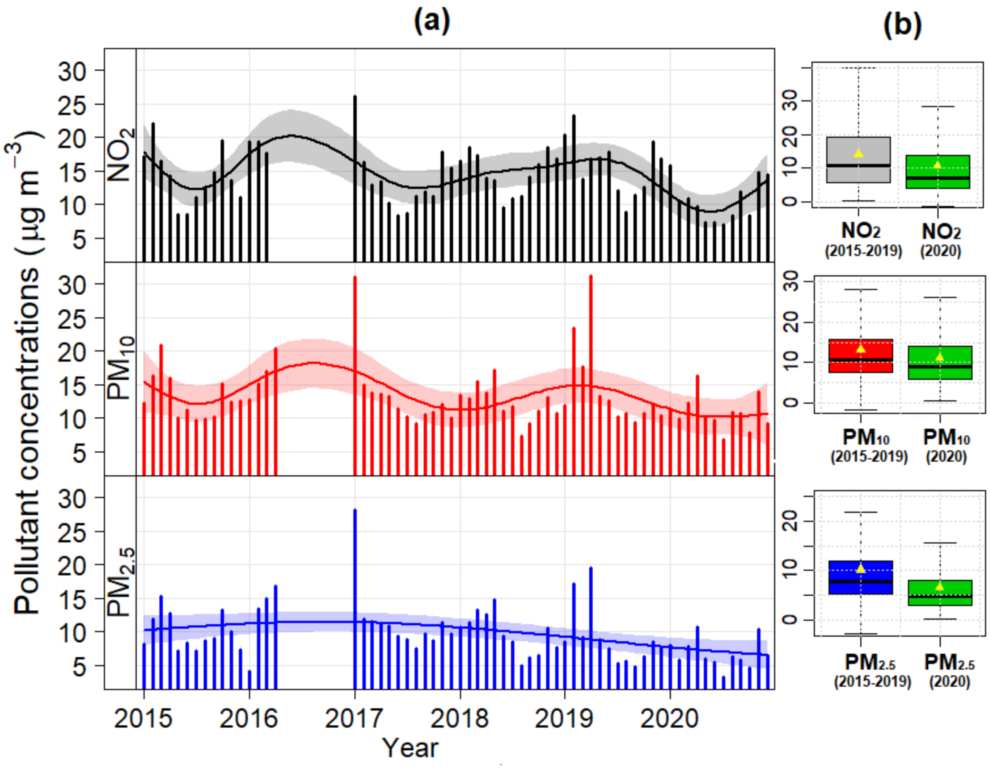

Figure 2 (a) Time-series (2015 – 2020) of monthly mean observed pollutant (NO_2, PM_{10} and $PM_{2.5}$) concentrations at urban background site (St Ebbe's) in Oxford, where shaded lines represent the smooth fit at the 95% confidence interval. Here right panel presents (b) box plots of the daily averaged air pollutant concentrations in 2020 versus 2015 – 2019.

588x453mm (38 x 38 DPI)

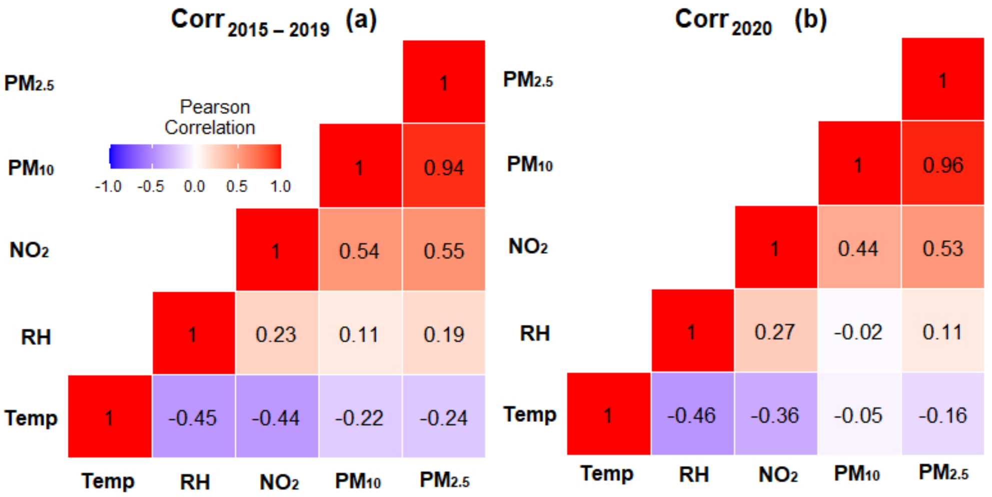

Figure 3 Correlation between daily mean air pollutant concentrations and meteorological variables during a) Covid-19 pandemic year (Corr2020) and b) pre-pandemic years (Corr$_{2015 - 2019}$) in Oxford.

521x271mm (38 x 38 DPI)

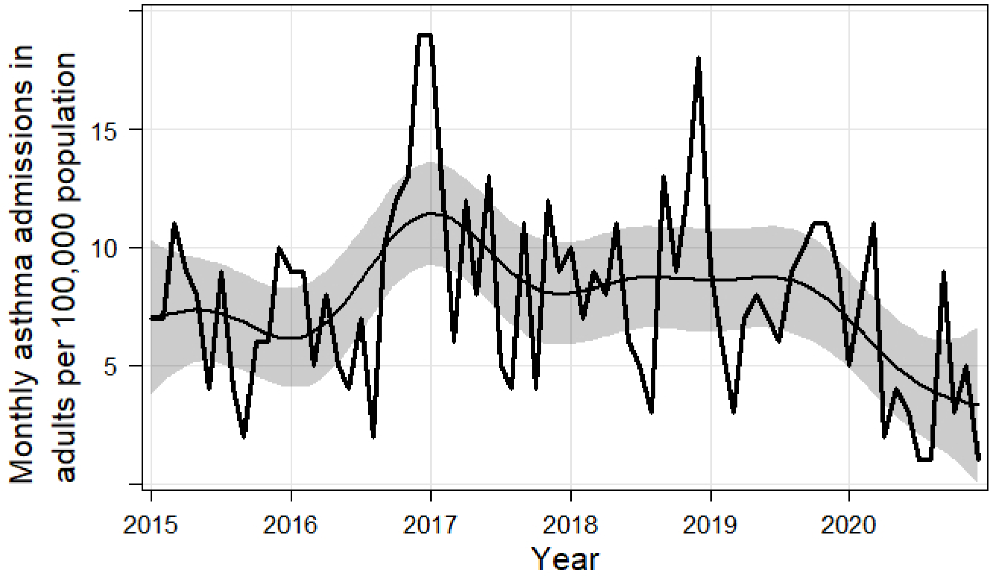

Figure 4 Time series of monthly adult asthma admissions, where the shaded lines represent the smooth fit at the 95% confidence interval.

439x259mm (38 x 38 DPI)

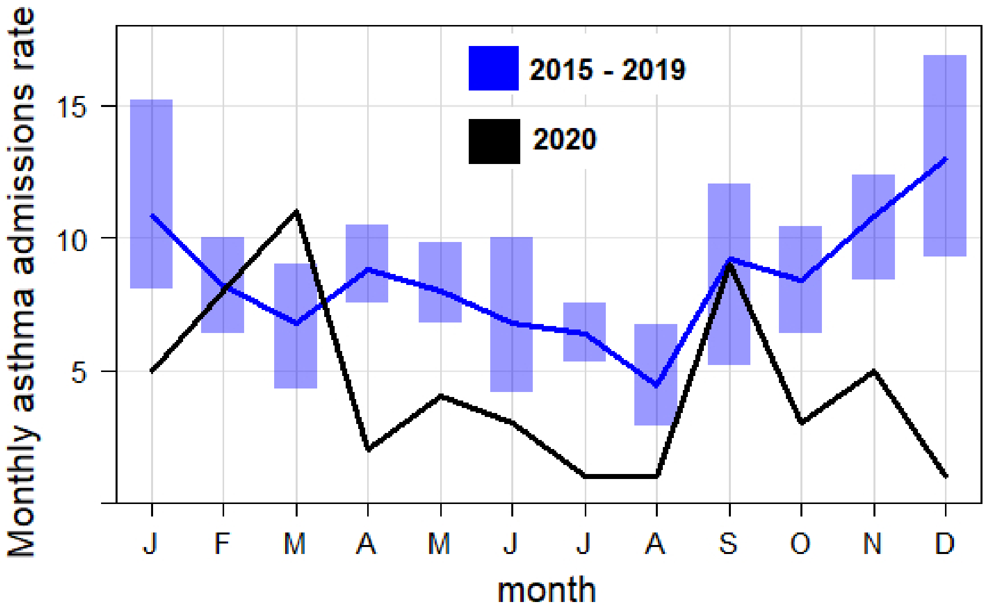

Figure 5 Mean monthly rate of adult asthma admissions in Oxford asthma in 2020 versus a five-year average. The shaded boxes represent the 95% confidence interval.

374x243mm (38 x 38 DPI)

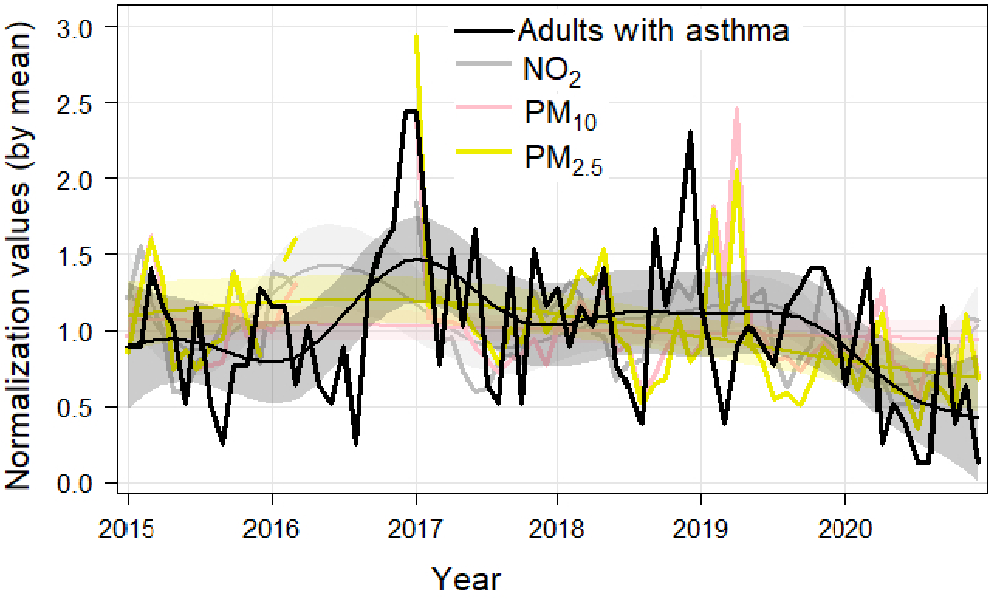

Figure 6 Normalized time series of monthly asthma admissions with air pollutants during 2015 – 2020. The shaded lines represent the smooth fit at the 95% confidence interval.

431x262mm (38 x 38 DPI)

STROBE Statement—checklist of items that should be included in reports of observational studies

	Item No	Recommendation
**Title and abstract**	1	(*a*) Indicate the study's design with a commonly used term in the title or the abstract (Page 2)
		(*b*) Provide in the abstract an informative and balanced summary of what was done and what was found (Pages 2, 3)
**Introduction**		
Background/rationale	2	Explain the scientific background and rationale for the investigation being reported (Pages 4, 5)
Objectives	3	State specific objectives, including any prespecified hypotheses (Page 5)
**Methods**		
Study design	4	Present key elements of study design early in the paper (Page 6)
Setting	5	Describe the setting, locations, and relevant dates, including periods of recruitment, exposure, follow-up, and data collection (Page 6)
Participants (Page 6)	6	(*a*) *Cohort study*—Give the eligibility criteria, and the sources and methods of selection of participants. Describe methods of follow-up<br>*Case-control study*—Give the eligibility criteria, and the sources and methods of case ascertainment and control selection. Give the rationale for the choice of cases and controls<br>*Cross-sectional study*—Give the eligibility criteria, and the sources and methods of selection of participants
		(*b*) *Cohort study*—For matched studies, give matching criteria and number of exposed and unexposed<br>*Case-control study*—For matched studies, give matching criteria and the number of controls per case
Variables	7	Clearly define all outcomes, exposures, predictors, potential confounders, and effect modifiers. Give diagnostic criteria, if applicable (Page 6, 7)
Data sources/ measurement	8*	For each variable of interest, give sources of data and details of methods of assessment (measurement). Describe comparability of assessment methods if there is more than one group (Pages 6, 7)
Bias	9	Describe any efforts to address potential sources of bias (NA)
Study size	10	Explain how the study size was arrived at (Page 7)
Quantitative variables	11	Explain how quantitative variables were handled in the analyses. If applicable, describe which groupings were chosen and why (Pages 7, 8)
Statistical methods (Pages 7, 8)	12	(*a*) Describe all statistical methods, including those used to control for confounding
		(*b*) Describe any methods used to examine subgroups and interactions
		(*c*) Explain how missing data were addressed
		(*d*) *Cohort study*—If applicable, explain how loss to follow-up was addressed<br>*Case-control study*—If applicable, explain how matching of cases and controls was addressed<br>*Cross-sectional study*—If applicable, describe analytical methods taking account of sampling strategy
		(*e*) Describe any sensitivity analyses

Continued on next page

For peer review only

Results		
Participants (Pages 9,10,11)	13*	(a) Report numbers of individuals at each stage of study—eg numbers potentially eligible, examined for eligibility, confirmed eligible, included in the study, completing follow-up, and analysed
		(b) Give reasons for non-participation at each stage
		(c) Consider use of a flow diagram
Descriptive data (Pages 9-11)	14*	(a) Give characteristics of study participants (eg demographic, clinical, social) and information on exposures and potential confounders
		(b) Indicate number of participants with missing data for each variable of interest
		(c) *Cohort study*—Summarise follow-up time (eg, average and total amount)
Outcome data	15*	*Cohort study*—Report numbers of outcome events or summary measures over time
		Case-control study—Report numbers in each exposure category, or summary measures of exposure
		Cross-sectional study—Report numbers of outcome events or summary measures
Main results (Pages 8-12)	16	(*a*) Give unadjusted estimates and, if applicable, confounder-adjusted estimates and their precision (eg, 95% confidence interval). Make clear which confounders were adjusted for and why they were included
		(*b*) Report category boundaries when continuous variables were categorized
		(*c*) If relevant, consider translating estimates of relative risk into absolute risk for a meaningful time period
Other analyses	17	Report other analyses done—eg analyses of subgroups and interactions, and sensitivity analyses (Page 12)
**Discussion**		
Key results	18	Summarise key results with reference to study objectives (Pages 12-14)
Limitations	19	Discuss limitations of the study, taking into account sources of potential bias or imprecision. Discuss both direction and magnitude of any potential bias (Page 14)
Interpretation	20	Give a cautious overall interpretation of results considering objectives, limitations, multiplicity of analyses, results from similar studies, and other relevant evidence (Page 14)
Generalisability	21	Discuss the generalisability (external validity) of the study results (Pages 12-14)
**Other information**		
Funding	22	Give the source of funding and the role of the funders for the present study and, if applicable, for the original study on which the present article is based (Page 16)

*Give information separately for cases and controls in case-control studies and, if applicable, for exposed and unexposed groups in cohort and cross-sectional studies.

Note: An Explanation and Elaboration article discusses each checklist item and gives methodological background and published examples of transparent reporting. The STROBE checklist is best used in conjunction with this article (freely available on the Web sites of PLoS Medicine at http://www.plosmedicine.org/, Annals of Internal Medicine at http://www.annals.org/, and Epidemiology at http://www.epidem.com/). Information on the STROBE Initiative is available at www.strobe-statement.org.

For peer review only

BMJ Open

Impacts of ambient air quality on acute asthma hospital admissions during the COVID-19 pandemic in Oxford City, UK; a time series study

Journal:	*BMJ Open*
Manuscript ID	bmjopen-2022-070704.R1
Article Type:	Original research
Date Submitted by the Author:	01-Sep-2023

Complete List of Authors:	Singh, Ajit; University of Birmingham, School of Geography Earth and Environmental Sciences; University of Birmingham, Institute of Applied Health Research<br>Morley, Gabriella L.; University of Birmingham, Institute of Microbiology and Infection<br>Coignet, Cécile; NHS Oxfordshire CCG<br>Leach, Felix; University of Oxford, Department of Engineering Science<br>Pope, Francis; University of Birmingham, Geography Earth and Environmental Sciences<br>Neil Thomas, G.; University of Birmingham, Institute of Applied Health Research<br>Stacey, Brian; Ricardo-AEA Ltd<br>Bush, Tony; University of Oxford, Department of Engineering Science; Apertum<br>Cole, Stuart; Oxfordshire County Council<br>Economides, George; Oxfordshire County Council<br>Anderson, Ruth; Oxfordshire County Council<br>Abreu, Pedro; Oxford City Council<br>Bartington, Suzanne; University of Birmingham, Institute of Applied Health Research

**Primary Subject Heading**:	Public health
Secondary Subject Heading:	Epidemiology
Keywords:	Asthma < THORACIC MEDICINE, COVID-19, EPIDEMIOLOGIC STUDIES, Epidemiology < THORACIC MEDICINE, Health policy < HEALTH SERVICES ADMINISTRATION & MANAGEMENT

SCHOLARONE™
Manuscripts

I, the Submitting Author has the right to grant and does grant on behalf of all authors of the Work (as defined in the below author licence), an exclusive licence and/or a non-exclusive licence for contributions from authors who are: i) UK Crown employees; ii) where BMJ has agreed a CC-BY licence shall apply, and/or iii) in accordance with the terms applicable for US Federal Government officers or employees acting as part of their official duties; on a worldwide, perpetual, irrevocable, royalty-free basis to BMJ Publishing Group Ltd ("BMJ") its licensees and where the relevant Journal is co-owned by BMJ to the co-owners of the Journal, to publish the Work in this journal and any other BMJ products and to exploit all rights, as set out in our licence.

The Submitting Author accepts and understands that any supply made under these terms is made by BMJ to the Submitting Author unless you are acting as an employee on behalf of your employer or a postgraduate student of an affiliated institution which is paying any applicable article publishing charge ("APC") for Open Access articles. Where the Submitting Author wishes to make the Work available on an Open Access basis (and intends to pay the relevant APC), the terms of reuse of such Open Access shall be governed by a Creative Commons licence – details of these licences and which Creative Commons licence will apply to this Work are set out in our licence referred to above.

Other than as permitted in any relevant BMJ Author's Self Archiving Policies, I confirm this Work has not been accepted for publication elsewhere, is not being considered for publication elsewhere and does not duplicate material already published. I confirm all authors consent to publication of this Work and authorise the granting of this licence.

For peer review only

Impacts of ambient air quality on acute asthma hospital admissions during the COVID-19 pandemic in Oxford City, UK; a time series study

Ajit Singh [a, b, †], Gabriella L. Morley [c, †], Cécile Coignet [d], Felix Leach [e], Francis D. Pope [a], Neil Thomas [b], Brian Stacey [f], Tony Bush [e], Stuart Cole [g], George Economides [g], Ruth Anderson [g], Pedro Abreu [h] and Suzanne E. Bartington [b, *]

[a] School of Geography, Earth and Environmental Sciences, University of Birmingham, Edgbaston Park Road, Birmingham, B15 2TT, UK; a.singh.2@bham.ac.uk ; f.pope@bham.ac.uk

[b] Institute of Applied Health Research, University of Birmingham, Edgbaston Park Road, Birmingham, B15 2TT, UK; s.bartington@bham.ac.uk; g.n.thomas@bham.ac.uk

[c] Institute of Microbiology and Infection, University of Birmingham, Edgbaston Park Road, Birmingham, B15 2TT, UK; GLM092@student.bham.ac.uk

[d] NHS Oxfordshire Clinical Commissioning Group, Jubilee House, 5510 John Smith Drive, Oxford Business Park South, Oxford, OX4 2LH; cecile.coignet@nhs.nete

[e] Department of Engineering Science, University of Oxford, Parks Road, Oxford, OX1 3PJ, UK; felix.leach@eng.ox.ac.uk ; tony.bush@apertum.co.uk

[f] Ricardo Energy & Environment, Gemini Building, Fermi Avenue, Harwell, Oxfordshire, OX11 0QR. Email: brian.stacey@ricardo.com

[g] Oxfordshire County Council, County Hall, New Road, Oxford, OX1 1ND, UK; stuart.cole@oxfordshire.gov.uk; George.economides@oxfordshire.gov.uk; ruth.anderson@oxfordshire.gov.uk

[h] Oxford City Council, Town Hall, St Aldate's, Oxford, OX1 1BX; pabreu@oxford.gov.uk

†Joint first authors

*Correspondence to: s.bartington@bham.ac.uk (S.E.B)

For peer review only

Abstract

Objectives To investigate the short-term associations between exposure to ambient air pollution (NO_2, $PM_{2.5}$ and PM_{10}) and incidence of asthma hospital admissions among adults, in Oxford, UK.

Design Retrospective time-series study

Setting Oxford City (postcode areas OX1 – OX4), UK.

Participants Adult population living within the postcode areas OX1 - OX4 in Oxford, UK from 1 January 2015 to 31 December 2021

Primary and Secondary outcome measures Hourly NO_2, $PM_{2.5}$ and PM_{10} concentrations and meteorological data for the period 1 January 2015 to 31 December 2020 were analyzed and used as exposures. We used Poisson linear regression analysis to identify independent associations between air pollutant concentrations and asthma admissions rate among the adult study population, using both single (NO_2, $PM_{2.5}$, PM_{10}) and multi-pollutant (NO_2 and $PM_{2.5}$, NO_2 and PM_{10}) models, where they adjustment for temperature and relative humidity.

Results The overall five-year average asthma admissions rate was 78 per 100,000 population during the study period. The annual average rate decreased to 46 per 100,000 population during 2020 (IRR 0.58, 95% CI 0.42 to 0.81, p <0.001) compared to the pre-pandemic years (2015 – 2019). In single pollutant analysis we observed a significantly increased risk of asthma admission associated with each 1 μg m^{-3} increase in monthly concentrations of NO_2 4% (95% CI 1.009 to 1.072), $PM_{2.5}$ 3% (95% CI 1.006 – 1.052) and PM_{10} 1.8% (95% CI 0.999 – 1.038). However, in the multi-pollutant regression model, the effect of each individual pollutant was attenuated.

Conclusions Ambient NO_2 and $PM_{2.5}$ air pollution exposure increased the risk of asthma admissions in this urban setting. Improvements in air quality during COVID-19 lockdown periods may have contributed to a substantially reduced acute asthma disease burden. Large-scale measures to improve air quality have potential to protect vulnerable people living with chronic asthma in urban areas.

For peer review only - http://bmjopen.bmj.com/site/about/guidelines.xhtml

Article Summary

Strengths and limitations of this study

- This study investigated the association between ambient air pollution exposure and risk of admission to hospital for asthma
- The study includes an at-risk population of >800,000 person years
- Associations were evaluated using single and multi-pollutant models
- Causation cannot be inferred from this observational study
- A single city study may limits wider generalisation of findings

For peer review only

For peer review only - http://bmjopen.bmj.com/site/about/guidelines.xhtml

1. Introduction and background

Asthma is a non-communicable chronic disease characterized by airway hyper-responsiveness, reversible airflow obstruction and inflammation.[1] As the most common respiratory condition worldwide, asthma affects more than 300 million people globally including 12% of the population in the UK[2] with 4.3 million UK adults requiring treatment for the condition.[3] In 2019 asthma contributed to 368 Years Lived in Disability (YLD) (95% Confidence Interval (CI): 238–546) and 410 per 100,000 DALYs (95% CI: 280–588)[4] among the UK population, with the greatest disease burden occurring in areas of high deprivation.[3] Asthma can also be fatal and approximately 1200 people a year die from asthma in the UK.[5] As there is no permanent cure, clinical management is directed towards preventing or reducing the severity of exacerbations and controlling symptoms including wheeze, cough and breathlessness.[6, 7] Primary prevention of asthma morbidity and mortality is therefore of major public health significance.

Asthma has multifactorial aetiology and is likely the result of complex gene and environmental interactions.[8] Environmental triggers may exacerbate symptoms so chronic asthma patients are advised to modify lifestyle and behaviours to reduce their exposure as a method for preventing exacerbations, alongside the use of inhaled or oral medication.[6] In recent years there has been increasing concern regarding the contribution of poor air quality to disease morbidity and mortality. Almost all of the world's population live in areas which do not meet 2021 WHO Global Air Quality Guidelines.[9-11] In the UK pollutants of major health concern include nitrogen dioxide (NO_2) which can cause airway inflammation, and fine particulate matter pollution – particles with diameter < 2.5 μm ($PM_{2.5}$), which can enter the circulation, with long term exposure to both pollutants linked to cardio-respiratory disease.[12-15] People with existing chronic respiratory diseases such as asthma or chronic obstructive pulmonary disease (COPD) are more vulnerable to $PM_{2.5}$ particle and NO_2 exposure[16] thereby exacerbating existing health inequalities. Long-term exposure to fine particulate matter ($PM_{2.5}$) and nitrogen dioxide (NO_2) is recognised to be causally associated with increased risk of developing child and adult asthma.[17, 18] Mechanisms linking air pollution exposure to asthma include modification of inflammatory pathways, leading to airway remodeling and enhancement of sensitivity to environmental allergens.[19] A recent meta-analysis of 22 studies undertaken in 12 countries showed a significant

For peer review only

For peer review only - http://bmjopen.bmj.com/site/about/guidelines.xhtml

association between ambient air pollution exposure (NO_2, $PM_{2.5}$, CO and O_3) and risk of asthma exacerbations among adults and children. A five-year time series study (2009 – 2013) across primary care settings in London identified that an increase in short-term exposure to NO_2 and PM_{10} was associated with a significant increase in respiratory consultations, inhaler prescriptions, or both.[20]

Improvements in ambient air quality have also been linked to reduced asthma morbidity. Implementation of emergency public health measures in response to the COVID-19 pandemic, led to substantive reductions in industrial and transportation activities from spring 2020 and related changes in air pollution levels.[21-23] Studies in major cities worldwide indicated lockdown periods were associated with a reduction in major air pollutant (NO_2, PM_{10} and $PM_{2.5}$) concentrations.[24-29] Studies in the UK revealed a similar pattern to global trends in terms of air quality changes,[22, 24, 30] most notably NO_2 reductions in urban areas.[21]

The outbreak of COVID-19 led to concerns that this viral pandemic would increase the number of asthma exacerbations, resulting in an influx of emergency department visits and hospitalisations among asthma patients. However, a national analysis of anonymised patient records in Scotland and Wales identified substantial reductions in severe asthma exacerbations leading to hospital admission during COVID-19 lockdown periods, although mortality rates remained unchanged.[31] A reduction in attendance to primary care for asthma exacerbations was also identified in a large-scale analysis of routine data in England.[32] Similar reductions in asthma emergency attendances and admissions among children were also identified across 15 countries worldwide,[33] suggested to be due to reduced exposure triggers and increased treatment adherence. Whilst it is likely that the trends described in air pollution are replicated in smaller cities and towns, the majority of existing research has been conducted in major cities[24, 30] with little opportunity to understand the relationship between changes in ambient air quality and asthma admissions among adults in smaller towns and cities in which 45% of the UK population live.[34]

In this study, a time-series analysis was conducted to investigate the association between ambient air pollution exposure and risk of unplanned (emergency) admissions among adults with asthma living in Oxford, UK.

For peer review only - http://bmjopen.bmj.com/site/about/guidelines.xhtml

2. Materials and methods

This study is part of the Natural Environment Research Council (NERC) funded OxAria Study' that aims to understand the impacts of COVID-19 on air and noise quality and associated health impacts in Oxford City (https://oxaria.org.uk/).

2.1 Study design

This study uses a retrospective time-series ecological design at the city level. The unit of analysis was a monthly count of the hospital admissions for adults, admitted with a primary diagnosis of acute asthma, during the study period of 1 January 2015 – 31 December 2020. Acute asthma is a serious condition involving progressive worsening of asthma symptoms, including breathlessness, wheeze, cough and chest tightness. Patients with severe of life-threatening acute asthma require immediate hospital treatment.

For peer review only

2.2 Setting – Oxford is a small historical city with an area of 46 km^2 and 68 m above sea level.[35] Oxford has a warm and temperate climate with mean annual temperature 10.3°C, mean annual cumulative rainfall 708 mm. The diverse population of this international city is approximately 152000, of which 79.6% are adults.[34] The city has two universities with approximately 34000 students enrolled. The city is a major centre for employment with a thriving economy, with an estimated 46000 people usually coming to the city for work, on a daily basis, before the COVID-19 pandemic.[36] The population is served by Oxford University Hospitals NHS Foundation Trust and comes under the jurisdiction of the Oxfordshire Clinical Commissioning Group (OCCG). A recent study by the OxAria team provides a detailed geographical, meteorological and environmental description of the study setting.[22]

***2.3* Study population** – The study population is adults aged >18 years living within Oxford City postcode areas OX1 – OX4 (see **Supplementary File – Figure S1**).

2.4 Data collection

2.4.1 Health outcome data

Hospital Episode Statistics (HES) data was accessed through OCCG. The study sample were obtained from weekly aggregated HES admissions data to a single tertiary hospital (John Radcliffe Hospital, Oxford) where all patients admitted with a primary diagnosis of asthma, as the outcome of interest, were included. Cases were

extracted based on the inclusion criteria of age (>18 years), residential postcode district (OX1 – OX4) and Clinical Classifications identifier: ICD-10 (International Classification of Diseases 10_{th} Revision code) coding for acute asthma (ICD-10, J45 to J46).[37] Exclusion criteria were all cases of wheeze (ICD-10, R06.2), cough (ICD-10, R05) and influenza (ICD-10, J11). Missing values within the HES outcome dataset were minimal (<4 %) over the total study duration. Here, missing values where present were replaced by interpolation based on the adjacent values. The mean monthly asthma hospital admissions rate was calculated and used as the unit of analysis.

2.4.2 Air pollution exposure and weather data

Hourly air pollutant concentrations (NO_2, $PM_{2.5}$ and PM_{10}) for the period 1 January 2015 to 31 December 2020 were obtained at the Urban and Rural Network (AURN) St Ebbe's urban background site (UKA00518),[38] located centrally in the OX1 – OX4 postcode area (covers 3-5 miles radius). Monthly mean air pollution concentrations were calculated from hourly datasets and analysed for the study period.

Hourly meteorological data including relative humidity (RH) and air temperature (T) from 1 January 2015 to 31 December 2020 were obtained from Oxford Radcliffe Observatory[39] – an urban background site, which was located in the OX2 postcode area. The proportion of missing hourly data was 16% (NO_2), 20% (PM_{10}) and 24% ($PM_{2.5}$) across the six–year study duration. This was not replaced with imputed data given the potential for fluctuations over this time-period which may result in an inaccuracy or bias.

2.5 COVID-19 lockdown classification

Data were obtained from 1 January 2015 to 31 December 2020. We consider two national lockdown periods in England in 2020: (i) Lockdown 1 (23 March – 15 June 2020) and (ii) Lockdown 2 (5 November – 2 December 2020).[22, 23]

2.6 Statistical analysis

Mean annual air pollutant concentrations (NO_2, $PM_{2.5}$, PM_{10}) for the study period (2015-2020) were calculated and compared to World Health Organisation Guideline levels.[11] Correlation between air pollutant concentrations and meteorological variables (air temperature and relative humidity) was explored using Pearson correlation matrices and co-efficient.

For peer review only - http://bmjopen.bmj.com/site/about/guidelines.xhtml

The incidence rate of asthma admissions was determined for each year and the incidence rate ratio (IRR) compared to 2019 (baseline year), with a comparison of 2020 admissions rate to a five-year average by Poisson regression analysis. A Pearson correlation matrix was used to compare asthma admissions and air pollutant concentrations for 2020 and pre-pandemic years (2015 – 2019). To compare the trends between asthma admissions and air pollutant concentrations, values normalized by the mean were calculated and a time-series was generated.

Poisson linear regression analysis was used to quantify independent associations between monthly air pollutant concentrations and incidence of asthma admissions among the adult study population, using single (NO_2, $PM_{2.5}$, PM_{10}) and multi-pollutant (NO_2 and $PM_{2.5}$, NO_2 and PM_{10}) models, with adjustment for temperature and relative humidity. All statistical analysis was conducted in STATA 15 applying 95% confidence intervals and two-tailed p-values at <0.05 considered statistically significant.[40]

2.7 Patient and Public Involvement

Public members were involved in the design and conduct of this study including public representation on the Study Steering Committee.

3. Results

3.1 Air quality

Overall mean annual average NO_2 and $PM_{2.5}$ concentrations for the total study duration exceeded the WHO Global Air Quality guideline average levels of 10 μg m^{-3} and 5 μg m^{-3}, for all study years respectively. A reduction in annual average NO_2, PM_{10} and $PM_{2.5}$ concentrations (0.74, 0.42 and 0.97 μg m^{-3} per year respectively) was observed from 2015 – 2019 which further reduced in 2020 (**Figure 1a**). There was also evidence of seasonal peaks with higher pollutant levels in the winter months for all three pollutants. The significant reductions in air pollutant concentrations in 2020 compared to 2015 - 2019 were attributed to Covid-19 national lockdown measures, primarily due to lower traffic emissions **(Figure 1b)**.[22]

It is observed that the mean NO_2 concentration reduced by 26.7% to 10.7 μg m^{-3} in 2020 (95% CI 8.7 to 12.7 μg m^{-3}) which was significantly (p<0.005) lower than the five-year average (2015 – 2019) concentration of 14.6 μg m^{-3} (95% CI 12.6 to 16.7 μg m-

For peer review only - http://bmjopen.bmj.com/site/about/guidelines.xhtml

^{3}). Similarly, the mean PM_{10} concentrations significantly ($p<0.05$) reduced by 18.6% in 2020 to 10.8 μg m^{-3} (95% CI 9.1 to 12.4 μg m^{-3}) compared with 13.2 μg m^{-3} (95% CI: 11.2 to 15.2 μg m^{-3}) from 2015 – 2019. Relatively, $PM_{2.5}$ levels showed a greater reduction than the other two pollutants, with mean $PM_{2.5}$ concentrations significantly ($p<0.005$) falling by 33.5% to 6.7 μg m^{-3} (95% CI 5.3 to 8.1 μg m^{-3}) in 2020 from 10.1 μg m^{-3} in 2015 – 2019 (95% CI 8.4 to 11.8 μg m^{-3}). However, a recent study using a machine learning for deweathering and detrending analysis has shown that these reductions were not only due to reduction in emissions but also influenced by the weather.[22]

$PM_{2.5}$ and PM_{10} levels were strongly positively correlated in both 2015-2019 (0.96) and 2020 (0.94) (**Supplementary File – Figure S2**). A weakly positive correlation was also observed for NO_2 and $PM_{2.5}$, higher temperatures were associated with lower levels of air pollution, with a negative correlation between all temperature and all three pollutants although this was weaker in 2020. Relative humidity had a weakly positive association throughout the study period.

3.2 Asthma admissions

The overall incidence of asthma admissions for the total study period was 7.8 per month per 100,000 population. This varied by season, with the highest admissions rate during winter and spring, and the lowest in the summer months (**Supplementary File – Figure S3**). We found that there were two notable peaks in admissions of adults with asthma in late 2016 and 2019 that coincided with peaks in PM concentrations (see **Figure 2**). Overall, the asthma admissions rate in 2020 was 42% lower than the five-year average (IRR 0.58, 95% CI 0.42 to 0.81, $p<0.001$) (Supplementary File – Table S1). There was no difference between the incidence of admissions in 2015 – 2018 compared with 2019, while there was a significantly reduced risk of admission in 2020 (IRR 0.6, $p<0.01$) compared to 2019 (**Table 1**).

Table 1 Annual Incidence of adult hospital admissions for acute asthma by year (2015-2020), compared with 2019 as baseline.

For peer review only - http://bmjopen.bmj.com/site/about/guidelines.xhtml

Year	Annual Incidence rate	IRR	95% CI	p value
**2015**	62.78	0.83	(0.61 – 1.11)	p >0.05
**2016**	79.00	1.04	(0.79 – 1.38)	p >0.05
**2017**	91.59	1.20	(0.92 – 1.58)	p >0.05
**2018**	84.20	1.12	(0.84 – 1.46)	p >0.05
**2019**	76.08	Baseline		
**2020**	45.70	0.60	(0.43 – 0.82)	p <0.01

*per 100,000 population

Further, **Figure 2** shows the monthly count of hospital admissions of adults with asthma in 2020 and compares with the monthly average pre-epidemic years (2015 – 2019). We found that the number of admissions for the whole first pandemic year after March 2020 was lower than the five-year average. Although, there was a similar pattern in summer 2020 admissions compared to the five-year average summer, where the admission rate remained lower. In contrast, a higher peak was noted in March 2020 compared to the previous five years. We also observe a peak in admissions during September, with admission rates nearly matching the five-year average.. There was a marked decline in admissions during the preceding months in 2020 which was not seen during the pre-pandemic years.

3.3 Understating the effect of air quality on asthma admissions in adults in response to COVID-19 measures in Oxford

Monthly mean normalized time series of air pollutant (NO_2, PM_{10} and $PM_{2.5}$) concentrations and asthma admissions are presented in **Figure 3**. Overall, variation in asthma admissions among adults mostly followed trends in air pollution over the six-year study period, where a positive association between air pollutants and asthma admissions was observed in 2015 – 2019 with a relatively stronger correlation than in 2020 (**Figure 3 and Supplementary File**). However, there was a negative correlation between air temperature and asthma admissions in 2020 and whereas a positive correlation was observed in pre-pandemic years (**Table S2**).

For peer review only - http://bmjopen.bmj.com/site/about/guidelines.xhtml

Further, to understand the association between air pollutants and asthma admissions among adults, single-pollutant and multi-pollutant models were generated applying Poisson regression analysis (**Table 2**). In the single-pollutant model, which was adjusted for temperature and relative humidity during the study period, showed that the risk of asthma admission increased significantly with increasing concentrations of NO_2 and $PM_{2.5}$. This was a 4% increase in risk for every 1 µg m^{-3} increase in mean monthly NO_2 and an approximately 3% increase in risk for every 1 µg m^{-3} increase in mean monthly $PM_{2.5}$. For every 1 µg m^{-3} increase in mean monthly PM_{10}, there was also a 1.8% increase in the risk of asthma admission, however, this failed to reach significance despite narrow confidence intervals.

Within the multi-pollutant model, the effect of each individual pollutant was non-significant. A weak correlation between pollutants makes it difficult for the model to separate the effects of correlated pollutants. Overall, in the multi-pollutants model we identified a non-significant 2.6% increase in risk of asthma admission per month predicted for every 1 µg m^{-3} increase in NO_2 and a non-significant 1.7% increase in risk of admission for every 1 µg m^{-3} increase $PM_{2.5}$, which approximates the risk for PM_{10}.

Table 2 Incidence rate ratio (IRR) for adult asthma admissions of in response to changes in air pollutant concentrations using the Poisson model.

Pollutants	Single-pollutant model			Multi-pollutant model		
	IRR	95% CI	p value	IRR	95% CI	p value
**NO_2**	1.040	1.009 – 1.072	p=0.012	1.026	0.987 – 1.067	p=0.188
**$PM_{2.5}$**	1.029	1.006 – 1.052	p=0.013	1.017	0.988 – 1.045	p=0.254
**PM_{10}**	1.018	0.999 – 1.038	p=0.07	NA	NA	NA
**Temperature**	0.98	0.950 – 1.020	p=0.340	0.99	0.960 – 1.030	p=0.620

For peer review only - http://bmjopen.bmj.com/site/about/guidelines.xhtml

**Relative Humidity**	1.01	0.990 – 1.020	p=0.466	1.00	0.990 – 1.020	p=0.826

4. Discussion

This study examines for the first time the effects of ambient air quality on asthma related hospital admissions in adults in Oxford city (UK), including impacts of the COVID-19 pandemic. A positive association was observed between risk of emergency asthma admission and air pollutant levels in Oxford, with hospital admission risk significantly increased even with modest increases in mean monthly NO_2 and $PM_{2.5}$ concentrations in single pollutant models. We also found a 42% reduction in the risk of acute asthma admission among adults living in Oxford in 2020 compared to the five-year average, suggesting air quality improvements during COVID-19 lockdown periods may have contributed to a reduction in severe asthma exacerbations. However, this observed association is in the context of a general reduction in emergency admissions for all causes during the COVID-19 period.

The findings of this work, supported by recent studies, found that pollution levels – notably NO_2 concentrations – decreased significantly in 2020 compared to pre-pandemic years[22, 23, 41, 42] due to changes in anthropogenic activities. This pattern needs to considered in the context of already declining levels of air pollution in Oxford and exceptional weather in 2020, including a warmer and windier early part of the year, coinciding with the first national lockdown.[22, 41] Recent studies by the OxAria team highlighted a clear reduction in traffic volume of 69% and 38% during the first and second lockdowns, respectively in Oxford, where NO_2 emissions from buses and cars reduced by 56% and 77% respectively.[22, 23] Whilst we did not attempt to explore contributions of specific emissions sources to changes in asthma admissions, the statistically significant effect of NO_2 and $PM_{2.5}$ in the single-pollutant model was not replicated in the multi-pollutant model, which indicates that changes in emissions sources may be relevant for determining overall asthma exacerbation risk in this context.

The findings presented in our study regarding the consistently positive association between NO_2 and $PM_{2.5}$ and asthma exacerbations rates are consistent with the

For peer review only

existing evidence.[43-52] The identified lockdown impact is also broadly consistent with a large-scale analysis of emergency admissions in Scotland and Wales which showed a 36% reduction in asthma related hospitalizations for all-ages population during the first 18 weeks of 2020,[31] however the fixed-effect meta-analysis model limited wider generalisability. Davies and colleagues proposed multiple possible theories for their observations, including reductions in NO_2 and $PM_{2.5}$ that were a national trend, however, unfortunately they were unable to substantiate this proposition as they did not collect concurrent air quality data. Further there exist differences in healthcare access and COVID-19 emergency public health regulation between the devolved nations and England. The first pandemic year, 2020 was also exceptional for the low rates of asthma admissions worldwide,[53] as previously reported in the paediatric population[54-58] and reflected in our own local admissions dataset.

There are other hypotheses for the most likely contributing factors to the reduction in asthma admissions in 2020, including speculation that patients were more vigilant with their asthma prescriptions and therefore had improved compliance to preventative inhaled steroid medication.[31] Given the fear about contracting COVID-19 when also diagnosed with a chronic condition[59] and the findings from the RECOVERY trial with adoption of steroids as a treatment for COVID-19,[60] it is plausible that patients improved compliance with their asthma plan, including compliance with any corticosteroid medication, during the pandemic and this contributed to a reduction in admissions during 2020.[31] This explanation has not been widely tested however, and it is unlikely that compliance continued throughout the entirety of 2020. An alternative theory is that many patients avoided healthcare services either due to fear that they may contract COVID-19 or a moral sense of duty to prevent overwhelming the NHS.[59, 61, 62] There is weak evidence though for patients with asthma delaying presentation given there was no change in asthma mortality and no increase in acuity of admissions from the studies which looked at this metric.[63, 64] This suggests that delays in seeking healthcare assistance was not a reason for the lower admission rates observed here. Furthermore, asthma admission was chosen instead of attendance for the outcome in this study to reduce the chance that the change observed was due to a change in patient behaviour (delay in presentation). Nevertheless, it would have been beneficial for this study and any future studies to

For peer review only - http://bmjopen.bmj.com/site/about/guidelines.xhtml

include a mechanism for measuring the extent to which a patient delayed presentation. Some studies have suggested that the low asthma admission rate may be due to low person-to-person transmission of other respiratory viruses, however this is unlikely to completely explain the dramatic reduction in 2020 admissions observed in multiple countries as well as the UK.[31, 61, 65, 66]

Strengths and limitations – This study has some additional limitations including the ecological, observational design that precludes the establishment of causality. Due to the observational study design it is difficult to conclude that changes in air pollution concentrations were wholly responsible for the reduction in asthma admissions during 2020. During the early pandemic phase several reports suggested that all-cause emergency admissions reduced, as the public responded to the “protect the NHS” campaign or were too fearful of coronavirus risks to present to hospital.[61] However, acute asthma is a severe condition which requires urgent hospital management and therefore it is likely that most patients in this study accessed emergency care following an acute attack. Our findings are also consistent with those of Shah and colleagues, who identified a statistically significant reduction in asthma patients who experienced an exacerbation during the lockdown period. The aggregated nature of the data collected on asthma admissions and the estimates of the population at risk means that caution must be exercised regarding any inferences at the individual level. Despite a defined population at risk, within a relatively small pollutant area, it is not known whether those admitted with asthma were exposed to the air pollutant concentrations measured. Further, asthma admissions data was aggregated to weekly time intervals due to the need for protection of patient anonymity; therefore restricting our capability to undertake daily analysis. We were also unable to include further seasonal influences (e.g., pollen exposure)[67] in the current study. Finally, we include data for ambient pollutant exposure only and recognise that adult patients with diagnosed asthma are likely to have spent a high duration of time indoors, particularly during lockdown periods. Indoor pollutants can have a significant impact on asthma conditions,[68] and it would be interesting to further investigate the potential impacts of indoor pollution on asthma admissions across the region. Further research is required to understand the respective changes in indoor and ambient pollutant concentrations during the COVID-19 pandemic and to better understand links to chronic disease outcomes among vulnerable patients. In particular, further studies

For peer review only - http://bmjopen.bmj.com/site/about/guidelines.xhtml

should analyse admissions at the patient-level to understand if pollutants have a particular impact on specific subgroups of the population with asthma which could be used to target future public health policies and guidance.

5. Conclusions

This study provides important insights into effects of changes in ambient air quality on asthma exacerbations in adults during the COVID-19 pandemic in Oxford, UK. The COVID-19 national lockdown measures have led to pronounced changes in air pollutant (NO_2, PM_{10} and $PM_{2.5}$) concentrations in Oxford. Our results show that adults with asthma had significantly lower risk of unplanned hospital admission in 2020 as compared to the pre-pandemic years (2015 – 2019). While this study cannot attribute exact causation, our findings suggest that improvements to air quality could reduce asthma healthcare service burden. There is a need to identify which pollutants, sources and locations would be most beneficial to target with future public health interventions to prevent severe asthma exacerbations requiring hospitalization in adults.

Author Contributions: S.E.B, F.L, F.D.P, G.N.T, B.S, T.B, S.C, G.E contributed to funding acquisition for the wider OxAria study. S.E.B, G.M and G.N.T conceived and designed the current study. R.A and C.C. acquired linked health data. T.B and P.A acquired air quality and meteorological data. G.M linked air quality and health data,

For peer review only - http://bmjopen.bmj.com/site/about/guidelines.xhtml

conducted initial data analysis and drafted the initial report under the supervision of S.E.B. A.S undertook data analysis, wrote the draft article, and undertook data visualisation. All authors contributed to editing and critically revising the article and approved the final draft. S.E.B is guarantor of the work.

Acknowledgments We are grateful to staff at Oxfordshire Clinical Commissioning Group, Oxfordshire County Council and Oxford City Council for supporting this work.

Funding This research was funded by the Natural Environment Research Council (grant no. NE/V010360/1). Its forerunning pilot project was funded by the National Institute for Health and Care Research (NIHR130095; NIHR Public Health Research). The views expressed are those of the author(s) and not necessarily those of the NIHR or the Department of Health and Social Care.

Data Availability Statement: Air quality and meteorology data are available in a public, open access repository. The hourly data for air quality are available on https://uk-air.defra.gov.uk. The hourly meteorology data are retrieved from the Oxford Radcliffe Observatory https://www.geog.ox.ac.uk/research/climate/rms/daily-data.html.

Conflicts of Interest: The authors declare no conflict of interest.

Ethical statement Ethical approval for the OxAria study was provided by the University of Birmingham Research Ethics Committee (ERN_20-0831).

References

[1] Holgate ST, Thomas M. Chapter 7 - Asthma. In: O'Hehir RE, Holgate ST, Sheikh A, eds. Middleton's Allergy Essentials: Elsevier 2017:151-204.

[2] NICE. What is the prevalence of asthma? National Institute for Health and Care Excellence, London, United Kingdom,2022. https://cks.nice.org.uk/topics/asthma/background-information/prevalence/ (accessed 15 May 2022).

[3] ALU. Asthma UK, Asthma facts and statistics. Asthma + Lung UK, London, United Kingdom, 2022. https://www.asthmaandlung.org.uk/media-centre/ (accessed 10 April 2022).

For peer review only

[4] IHME. The global burden of disease study, Institute for Health Metrics and Evaluation, University of Washington, USA, 2022. https://vizhub.healthdata.org/gbd-compare/ (accessed 28 September 2022).
[5] BLF. Asthma statistics. British Lung Foundation, London, United Kingdom, 2022. https://statistics.blf.org.uk/asthma (accessed 15 May 2022).
[6] Hoffmann C, Maglakelidze M, Von SE, et al. Asthma and COPD exacerbation in relation to outdoor air pollution in the metropolitan area of Berlin, Germany. *Respiratory research* 2022; **23**(1)64. doi.org/10.1186/s12931-022-01983-1
[7] NICE. Scenario: Newly-diagnosed asthma. National Institute for Health and Care Excellence, London, United Kingdom, 2021. https://cks.nice.org.uk/topics/asthma/management/newly-diagnosed-asthma/ (accessed 15 May 2022).
[8] Subbarao P, Mandhane PJ, Sears MR. Asthma: epidemiology, etiology and risk factors. *Canadian Medical Association Journal* 2009;**181**(9):E181. doi:10.1503/cmaj.080612
[9] WHO. WHO's Urban Ambient Air Pollution Database–Update 2016. Geneva: World Health Organization, 2016. https://cdn.who.int/media/docs/default-source/air-quality-database/aqd-2016/aap_database_summary_results_2016_v02.pdf?sfvrsn=384beb23_3 (accessed 15 May 2022).
[10] WHO. Billions of people still breathe unhealthy air: new WHO data. World Health Organization, Geneva, Switzerland, 2022. https://www.who.int/news/item/04-04-2022-billions-of-people-still-breathe-unhealthy-air-new-who-data (accessed 15 May 2022)
[11] WHO. WHO global air quality guidelines: particulate matter ($PM_{2.5}$ and PM_{10}), ozone, nitrogen dioxide, sulfur dioxide and carbon monoxide. World Health Organization, Geneva, Switzerland, 2021. https://apps.who.int/iris/handle/10665/345329 (accessed 15 May 2022).
[12] Madureira J, Brancher EA, Costa C, et al. Cardio-respiratory health effects of exposure to traffic-related air pollutants while exercising outdoors: A systematic review. *Environmental Research* 2019;**178**:108647. doi:10.1016/j.envres.2019.108647
[13] PHE. Health matters: air pollution. Public Health England, London, United Kingdom, 2018. https://www.gov.uk/government/publications/health-matters-air-pollution/health-matters-air-pollution (accessed 15 May 2022)
[14] PHE. Review of interventions to improve outdoor air quality and public health. Public Health England, London, United Kingdom, 2019. https://assets.publishing.service.gov.uk/government/uploads/system/uploads/attachment_data/file/938623/Review_of_interventions_to_improve_air_quality_March-2019-2018572.pdf (accessed 15 May 2022).
[15] ONS. Road transport and air emissions. Office for National Statistics, London, United Kingdom, 2019. https://www.ons.gov.uk/economy/environmentalaccounts/articles/roadtransportandairemissions/2019-09-16 (accessed 15 May 2022).
[16] Jiang X-Q, Mei X-D, Feng D. Air pollution and chronic airway diseases: what should people know and do? *Journal of Thoracic Disease* 2015;**8**(1):E31-E40. doi:10.3978/j.issn.2072-1439.2015.11.50
[17] Jacquemin B, Siroux V, Sanchez M, et al. Ambient Air Pollution and Adult Asthma Incidence in Six European Cohorts (ESCAPE). *Environmental Health Perspectives* 2015;**123**(6):613-21. doi:10.1289/ehp.1408206

For peer review only - http://bmjopen.bmj.com/site/about/guidelines.xhtml

[18] Khreis H, Kelly C, Tate J, et al. Exposure to traffic-related air pollution and risk of development of childhood asthma: A systematic review and meta-analysis. *Environment International* 2017;**100**:1-31. doi:10.1016/j.envint.2016.11.012
[19] COMEAP. Committee on the Medical Effects of Air Pollutants. Centre for Radiation, Chemical and Environmental Hazards, Chilton, United Kingdom, 2022. https://www.gov.uk/government/groups/committee-on-the-medical-effects-of-air-pollutants-comeap (accessed 15 May 2022).
[20] Ashworth M, Analitis A, Whitney D, et al. Spatio-temporal associations of air pollutant concentrations, GP respiratory consultations and respiratory inhaler prescriptions: a 5-year study of primary care in the borough of Lambeth, South London. *Environmental Health* 2021;**20**(1):54. doi:10.1186/s12940-021-00730-1
[21] AQEG. Report: Estimation of changes in air pollution emissions, concentrations and exposure during the COVID-19 outbreak in the UK: Rapid evidence review. The Air Quality Expert Group, Department for Environment, Food and Rural Affairs (Defra), London, United Kingdom, 2020. https://uk-air.defra.gov.uk/assets/documents/reports/cat09/2007010844_Estimation_of_Changes_in_Air_Pollution_During_COVID-19_outbreak_in_the_UK.pdf (accessed 15 May 2022).
[22] Singh A, Bartington SE, Song C, et al. Impacts of emergency health protection measures upon air quality, traffic and public health: evidence from Oxford, UK. *Environmental Pollution* 2022;**293**:118584. doi:10.1016/j.envpol.2021.118584
[23] Singh A, Guo T, Bush T, et al. Impacts of COVID-19 lockdown on traffic flow, active travel and gaseous pollutant concentrations; implications for future emissions control measures in Oxford, UK. *Sustainability* 2022; **14**(23): 16182. doi.org/10.3390/su142316182
[24] Shi Z, Song C, Liu B, et al. Abrupt but smaller than expected changes in surface air quality attributable to COVID-19 lockdowns. *Science Advances* 2021;**7**(3):eabd6696. doi:10.1126/sciadv.abd6696
[25] Mahato S, Pal S, Ghosh KG. Effect of lockdown amid COVID-19 pandemic on air quality of the megacity Delhi, India. *Science of the Total Environment* 2020;**730**:139086. doi:10.1016/j.scitotenv.2020.139086
[26] Kumar P, Hama S, Omidvarborna H, et al. Temporary reduction in fine particulate matter due to 'anthropogenic emissions switch-off'during COVID-19 lockdown in Indian cities. *Sustainable cities and society* 2020;**62**:102382.
[27] Baldasano JM. COVID-19 lockdown effects on air quality by NO2 in the cities of Barcelona and Madrid (Spain). *Science of the Total Environment* 2020;**741**:140353. doi:10.1016/j.scs.2020.102382
[28] Lian X, Huang J, Huang R, et al. Impact of city lockdown on the air quality of COVID-19-hit of Wuhan city. *Science of the Total Environment* 2020;**742**:140556. doi:10.1016/j.scitotenv.2020.140556
[29] Liu F, Wang M, Zheng M. Effects of COVID-19 lockdown on global air quality and health. *Science of The Total Environment* 2021;**755**:142533. doi:10.1016/j.scitotenv.2020.142533
[30] Jephcote C, Hansell AL, Adams K, et al. Changes in air quality during COVID-19 'lockdown' in the United Kingdom. *Environmental Pollution* 2021;**272**:116011. doi:10.1016/j.envpol.2020.116011
[31] Davies GA, Alsallakh MA, Sivakumaran S, et al. Impact of COVID-19 lockdown on emergency asthma admissions and deaths: national interrupted time

For peer review only

series analyses for Scotland and Wales. *Thorax* 2021;**76**(9):867. doi: 10.1136/thoraxjnl-2020-216380

[32] Shah SA, Quint JK, Nwaru BI, et al. Impact of COVID-19 national lockdown on asthma exacerbations: interrupted time-series analysis of English primary care data. *Thorax* 2021;**76**(9):860. doi:10.1136/thoraxjnl-2020-216512

[33] Papadopoulos NG, Mathioudakis AG, Custovic A, et al. Childhood asthma outcomes during the COVID-19 pandemic: Findings from the PeARL multi-national cohort. *Allergy* 2021;**76**(6):1765-75. doi:10.1111/all.14787

[34] ONS. Population estimates for the UK, England and Wales, Scotland and Northern Ireland: mid-2019, using April 2019 local authority district codes. Office for National Statistics, Fareham, England, 2020. https://www.ons.gov.uk/peoplepopulationandcommunity/populationandmigration/populationestimates/datasets/populationestimatesforukenglandandwalesscotlandandnorthernireland (accessed 18 May 2021).

[35] OP AM. AM Online Projects. Oxford, United Kingdom, 2022. https://en.climate-data.org/europe/united-kingdom/england/oxford-22/ (accessed 18 May 2022).

[36] OCC. Oxford Profile 2018 Key Facts Oxford City Council. Oxford, United Kingdom, 2018. https://www.oxford.gov.uk/downloads/file/5021/oxford_profile_2018 (accessed 18 May 2022).

[37] ICD. International Statistical Classification of Diseases and Related Health Problems 10th Revision (ICD-10) Version for 2010. International Classification of Diseases, United Nations System, 2010. https://icd.who.int/browse10/2010/en (accessed 18 May 2022).

[dataset] [38] DEFRA. UK air information. Department for Environment Food & Rural Affairs, United Kingdom, April 10, 2022. https://uk-air.defra.gov.uk

[dataset] [39] SoGE. Daily data from the Radcliffe Observatory site in Oxford. School of Geography and Environment, University of Oxford, Oxford, United Kingdom, April 10, 2022. https://www.geog.ox.ac.uk/research/climate/rms/daily-data.html

[40] STATA. Stata 15: Statistical software for data science, 2022. https://www.stata.com/stata15/ (accessed 10 April 2022).

[41] Carslaw D. Blog update on COVID-19 and changes in air pollution. Ricardo, United Kingdom. https://ee.ricardo.com/news/blog-update-on-covid-19-and-changes-in-air-pollution (accessed 18 May 2022).

[42] Abreu P. Air Quality Annual Status Report. Oxford City Council, Oxford, United Kingdom, 2021. https://www.oxford.gov.uk/downloads/file/7612/air_quality_annual_status_report_2020 (accessed 18 May 2022).

[43] Orellano P, Quaranta N, Reynoso J, et al. Effect of outdoor air pollution on asthma exacerbations in children and adults: systematic review and multilevel meta-analysis. *PloS one* 2017;**12**(3):e0174050. doi:10.1371/journal.pone.0174050

[44] Guarnieri M, Balmes JR. Outdoor air pollution and asthma. *The Lancet* 2014;**383**(9928):1581-92. doi:10.1016/S0140-6736(14)60617-6

[45] Sunyer J, Spix C, Quénel P, et al. Urban air pollution and emergency admissions for asthma in four European cities: the APHEA Project. *Thorax* 1997;**52**(9):760. doi:10.1136/thx.52.9.760

[46] Muñoz X, Barreiro E, Bustamante V, et al. Diesel exhausts particles: Their role in increasing the incidence of asthma. Reviewing the evidence of a causal

For peer review only - http://bmjopen.bmj.com/site/about/guidelines.xhtml

link. *Science of The Total Environment* 2019, 652: 1129-1138. https://doi.org/10.1016/j.scitotenv.2018.10.188

[47] Wong KL, Wong WHS, Yau YS, et al.. Asthma admission among children in Hong Kong during the first year of the COVID-19 pandemic. *Pediatric Pulmonology* 2022, **57**(12): 3104-3110. doi:10.1002/ppul.26141

[48] Galán I, Tobías A, Banegas JR, et al. Short-term effects of air pollution on daily asthma emergency room admissions. *European Respiratory Journal* 2003;**22**(5):802. doi:10.1183/09031936.03.00013003

[49] Jacquemin B, Schikowski T, Carsin AE, et al. The Role of Air Pollution in Adult-Onset Asthma: A Review of the Current Evidence. *Seminars in Respiratory and Critical Care Medicine* 2012;**33**(06):606-19. doi:10.1055/s-0032-1325191

[50] Tiotiu AI, Novakova P, Nedeva D, et al. Impact of Air Pollution on Asthma Outcomes. *International Journal of Environmental Research and Public Health* 2020; **17**(17): 6212. doi:10.3390/ijerph17176212

[51] Kuo C-Y, Chan C-K, Wu C-Y, et al. The Short-Term Effects of Ambient Air Pollutants on Childhood Asthma Hospitalization in Taiwan: A National Study. *International Journal of Environmental Research and Public Health* 2019;**16**(2):203. doi:10.3390/ijerph16020203

[52] Lam HC-y, Li AM, Chan EY-y, et al. The short-term association between asthma hospitalisations, ambient temperature, other meteorological factors and air pollutants in Hong Kong: a time-series study. *Thorax* 2016;**71**(12):1097. doi:10.1136/thoraxjnl-2015-208054

[53] Onay ZR, Mavi D, Ayhan Y, et al. Did Hospital Admissions Caused by Respiratory Infections and Asthma Decrease During the COVID-19 Pandemic? *Medeniyet Medical Journal* 2022;**37**(1):92-98. doi:10.4274/MMJ.galenos.2022.02779

[54] Krivec U, Kofol Seliger A, Tursic J. COVID-19 lockdown dropped the rate of paediatric asthma admissions. *Archives of Disease in Childhood* 2020;**105**(8):809. doi:10.1136/archdischild-2020-319522

[55] Williams TC, MacRae C, Swann OV, et al. Indirect effects of the COVID-19 pandemic on paediatric healthcare use and severe disease: a retrospective national cohort study. *Archives of Disease in Childhood* 2021;**106**(9):911. doi:10.1136/archdischild-2020-321008

[56] Dann L, Fitzsimons J, Gorman KM, et al. Disappearing act: COVID-19 and paediatric emergency department attendances. *Archives of Disease in Childhood* 2020;**105**(8):810. doi:10.1136/archdischild-2020-319654

[57] Ashikkali L, Carroll W, Johnson C. The indirect impact of COVID-19 on child health. *Paediatrics and Child Health* 2020;**30**(12):430-37. doi:10.1016/j.paed.2020.09.004

[58] Vásquez-Hoyos P, Diaz-Rubio F, Monteverde-Fernandez N, et al. Reduced PICU respiratory admissions during COVID-19. *Archives of Disease in Childhood* 2021;**106**(8):808. doi:10.1136/archdischild-2020-320469

[59] Lazzerini M, Barbi E, Apicella A, et al. Delayed access or provision of care in Italy resulting from fear of COVID-19. *The Lancet Child & Adolescent Health* 2020;**4**(5):e10-e11. doi:10.1016/S2352-4642(20)30108-5

[60] Group TWREAfC-TW. Association Between Administration of Systemic Corticosteroids and Mortality Among Critically Ill Patients With COVID-19: A Meta-analysis. *JAMA* 2020;**324**(13):1330-41. doi:10.1001/jama.2020.17023

[61] Mulholland RH, Wood R, Stagg HR, et al. Impact of COVID-19 on accident and emergency attendances and emergency and planned hospital admissions in

For peer review only - http://bmjopen.bmj.com/site/about/guidelines.xhtml

Scotland: an interrupted time-series analysis. *Journal of the Royal Society of Medicine* 2020;**113**(11):444-53. doi:10.1177/0141076820962447
[62] TFLH. Over a third of people with lung conditions felt pressure to avoid or delay seeking treatment during lockdown London. Taskforce for Lung Health, London, United Kingdom, 2020. https://www.blf.org.uk/taskforce/press-release/over-a-third-of-people-with-lung-conditions-felt-pressure-to-avoid-or-delay-seeking-treatment (accessed 09 July 2022).
[63] Chavasse R, Almario A, Christopher A, et al. The Indirect Impact of COVID-19 on Children With Asthma. *Archivos de Bronconeumología* 2020;**56**(11):768-69. doi:10.1016/j.arbres.2020.07.003
[64] Sykes DL, Faruqi S, Holdsworth L, et al. Impact of COVID-19 on COPD and asthma admissions, and the pandemic from a patient's perspective. *ERJ Open Research* 2021;**7**(1):00822-2020. doi:10.1183/23120541.00822-2020
[65] Abe K, Miyawaki A, Nakamura M, et al. Trends in hospitalizations for asthma during the COVID-19 outbreak in Japan. *The Journal of Allergy and Clinical Immunology: In Practice* 2021;**9**(1):494-96.e1. doi:10.1016/j.jaip.2020.09.060
[66] Jones N. How coronavirus lockdowns stopped flu in its tracks. Nature news. *https://wwwnaturecom/articles/d41586-020-01538-8* (accessed 09 July 2022).
[67] Gonzalez-Barcala FJ, Aboal-Viñas J, Aira MJ, et al. Influence of Pollen Level on Hospitalizations for Asthma. *Archives of Environmental & Occupational Health* 2013, **68**(2): 66-71. doi.org/10.1080/19338244.2011.638950
[68] Carazo FL, Fernández AR, González-Barcala, FJ, et al. Indoor Air Contaminants and Their Impact on Respiratory Pathologies. *Archivos de Bronconeumología* (English Edition), **49**(1): 22-27. doi.org/10.1016/j.arbr.2012.11.004

Figure legends

Figure 1 (a) Time-series (2015 – 2020) of monthly mean observed pollutant (NO_2, PM_{10} and $PM_{2.5}$) concentrations at urban background site (St Ebbe's) in Oxford, where shaded lines represent the smooth fit at the 95% confidence interval. Here right panel presents **(b)** box plots of the daily averaged air pollutant concentrations in 2020 versus 2015 – 2019.

Figure 2 Mean monthly rate of adult asthma admissions in Oxford asthma in 2020 versus a five-year average. The shaded boxes represent the 95% confidence interval.

For peer review only - http://bmjopen.bmj.com/site/about/guidelines.xhtml

Figure 3 Normalized time series of monthly asthma admissions with air pollutants during 2015 – 2020. The shaded lines represent the smooth fit at the 95% confidence interval.

For peer review only

For peer review only - http://bmjopen.bmj.com/site/about/guidelines.xhtml

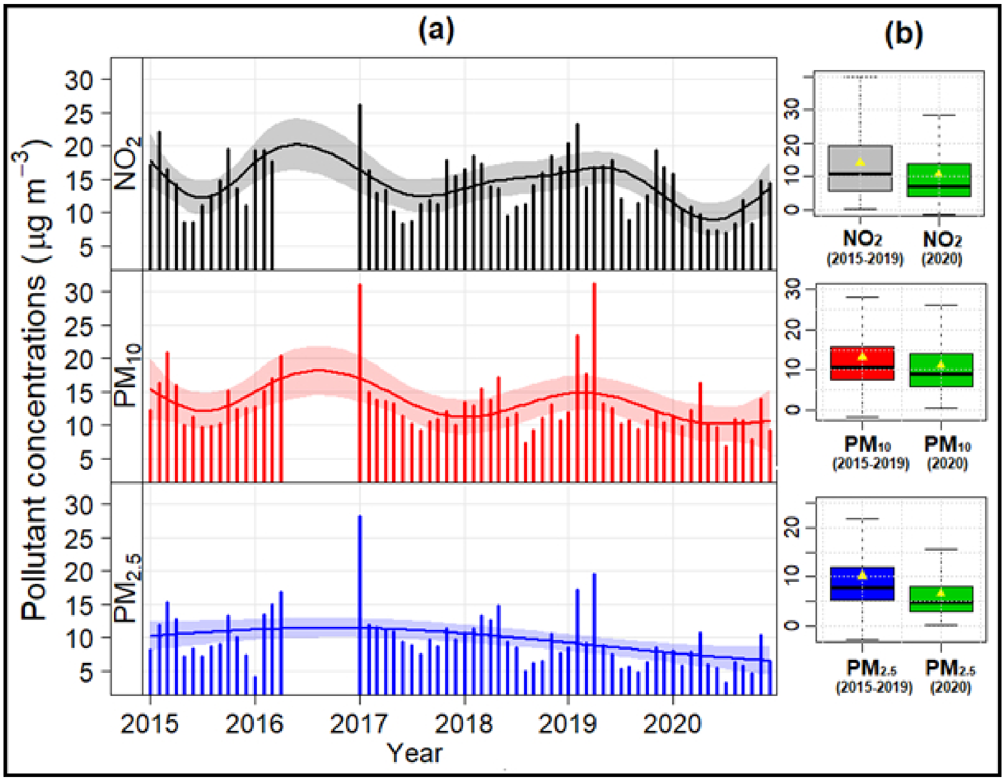

Figure 1 (a) Time-series (2015 – 2020) of monthly mean observed pollutant (NO2, PM10 and PM2.5) concentrations at urban background site (St Ebbe's) in Oxford, where shaded lines represent the smooth fit at the 95% confidence interval. Here right panel presents (b) box plots of the daily averaged air pollutant concentrations in 2020 versus 2015 – 2019.

405x312mm (38 x 38 DPI)

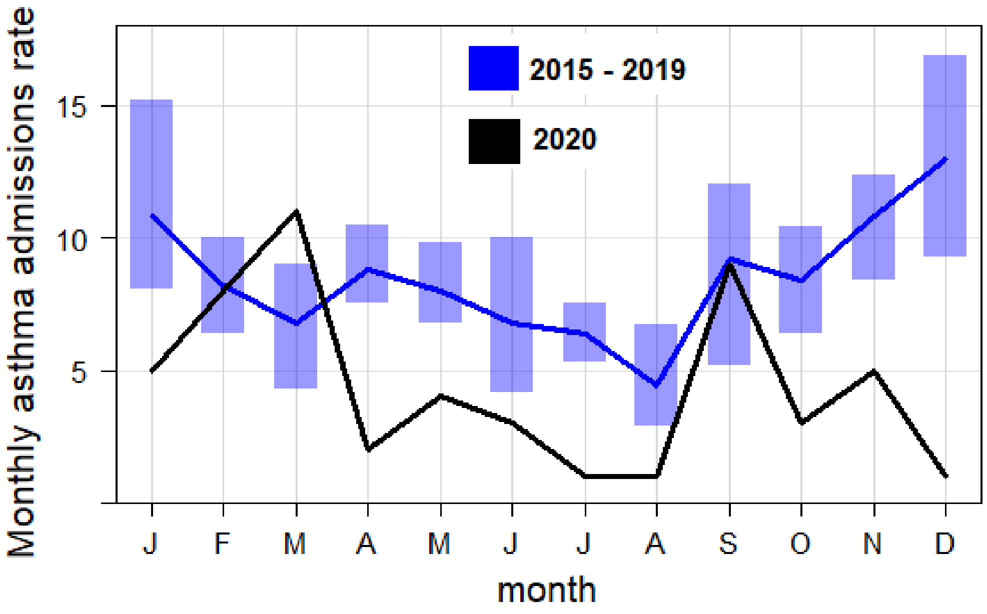

Figure 2 Mean monthly rate of adult asthma admissions in Oxford asthma in 2020 versus a five-year average. The shaded boxes represent the 95% confidence interval. In 2020, two major national Lockdown periods are considered, 23 March – 15 June 2020 and 5 November – 2 December 2020.

374x243mm (38 x 38 DPI)

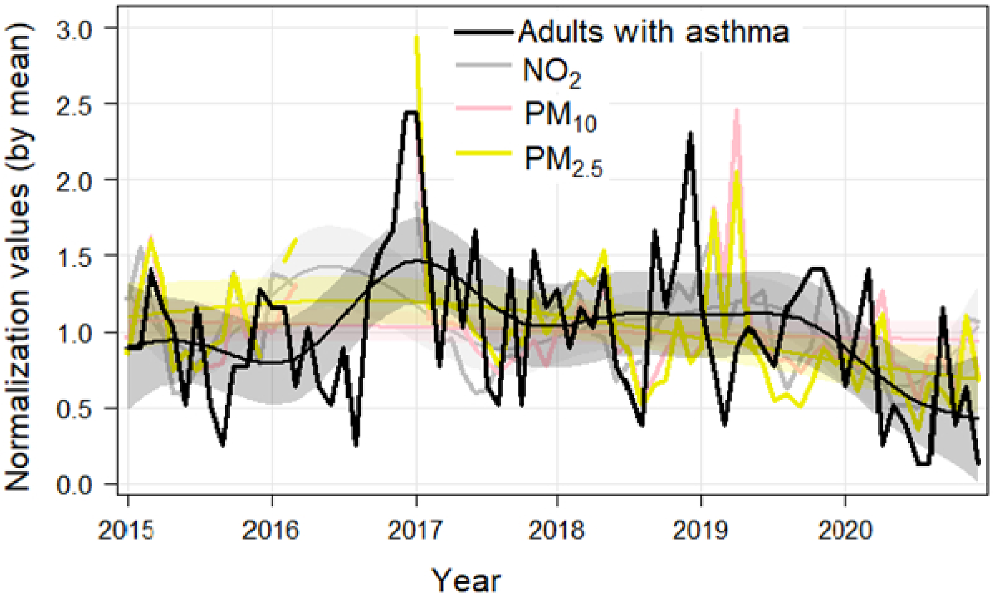

Figure 3 Normalized time series of monthly asthma admissions with air pollutants during 2015 – 2020. The shaded lines represent the smooth fit at the 95% confidence interval.

402x243mm (38 x 38 DPI)

Supplementary material:

Impacts of ambient air quality on acute asthma hospital admissions during the COVID-19 pandemic in Oxford City, UK; a time series study

Ajit Singh [a, b, †], Gabriella L. Morley [c, †], Cécile Coignet [d], Felix Leach [e], Francis D. Pope [a], Neil Thomas [b], Brian Stacey [f], Tony Bush [e], Stuart Cole [g], George Economides [g], Ruth Anderson [g], Pedro Abreu [h] and Suzanne E. Bartington [b, *]

[a] School of Geography, Earth and Environmental Sciences, University of Birmingham, Edgbaston Park Road, Birmingham, B15 2TT, UK; a.singh.2@bham.ac.uk ; f.pope@bham.ac.uk

[b] Institute of Applied Health Research, University of Birmingham, Edgbaston Park Road, Birmingham, B15 2TT, UK; s.bartington@bham.ac.uk; g.n.thomas@bham.ac.uk

[c] Institute of Microbiology and Infection, University of Birmingham, Edgbaston Park Road, Birmingham, B15 2TT, UK; GLM092@student.bham.ac.uk

[d] NHS Oxfordshire Clinical Commissioning Group, Jubilee House, 5510 John Smith Drive, Oxford Business Park South, Oxford, OX4 2LH; cecile.coignet@nhs.nete

[e] Department of Engineering Science, University of Oxford, Parks Road, Oxford, OX1 3PJ, UK; felix.leach@eng.ox.ac.uk ; tony.bush@apertum.co.uk

[f] Ricardo Energy & Environment, Gemini Building, Fermi Avenue, Harwell, Oxfordshire, OX11 0QR. Email: brian.stacey@ricardo.com

[g] Oxfordshire County Council, County Hall, New Road, Oxford, OX1 1ND, UK; stuart.cole@oxfordshire.gov.uk; George.economides@oxfordshire.gov.uk; ruth.anderson@oxfordshire.gov.uk

[h] Oxford City Council, Town Hall, St Aldate's, Oxford, OX1 1BX; pabreu@oxford.gov.uk

†Joint first authors

*Correspondence to: s.bartington@bham.ac.uk (S.E.B)

For peer review only

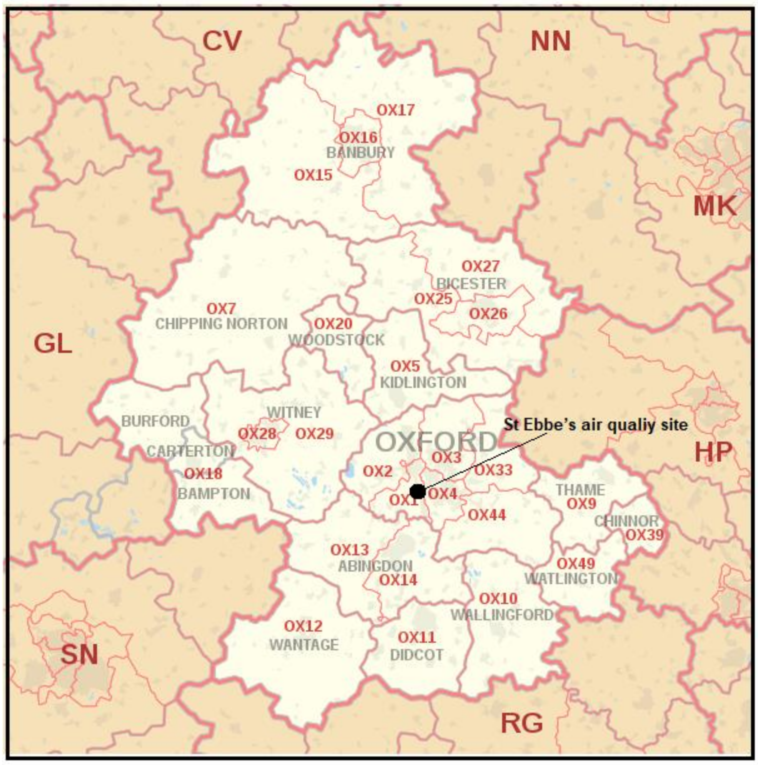

Figure S1 Map of Oxford postcode districts illustrating Oxford City in OX1, OX2, OX3 and OX4. (Source, https://en.wikipedia.org/wiki/File:OX_postcode_area_map.svg).

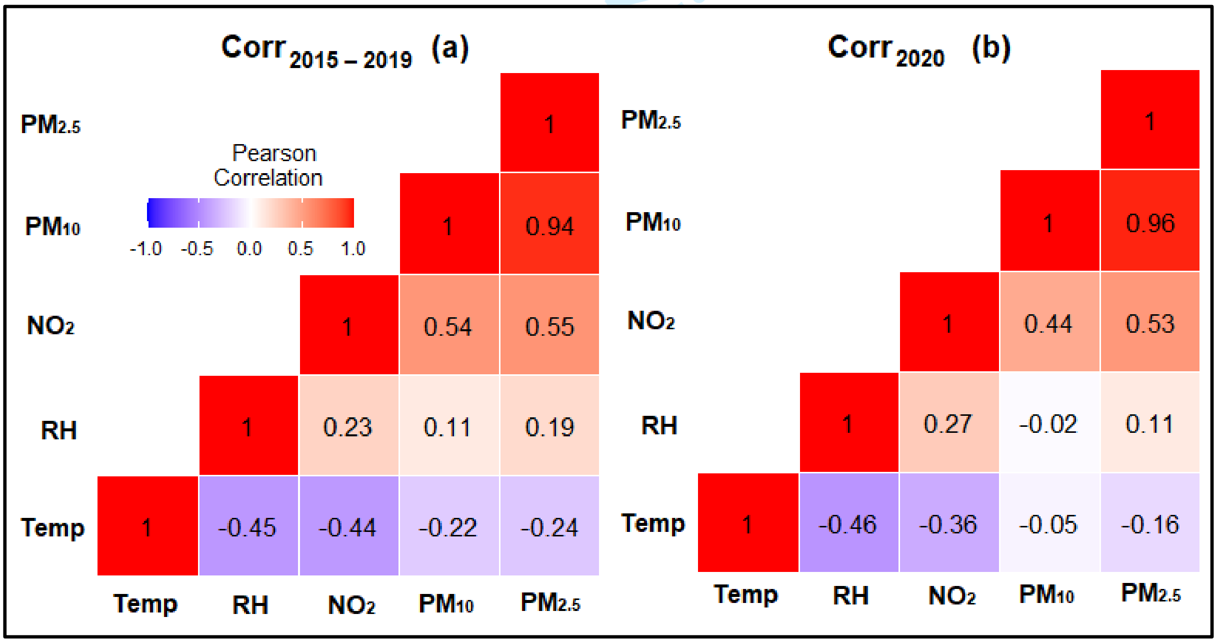

Figure S2 Correlation between daily mean air pollutant concentrations and meteorological variables during **a)** Covid-19 pandemic year (Corr*2020*) and **b)** pre-pandemic years (Corr*2015 – 2019*) in Oxford. Here, RH and Temp denote relative humidity and temperature, respectively.

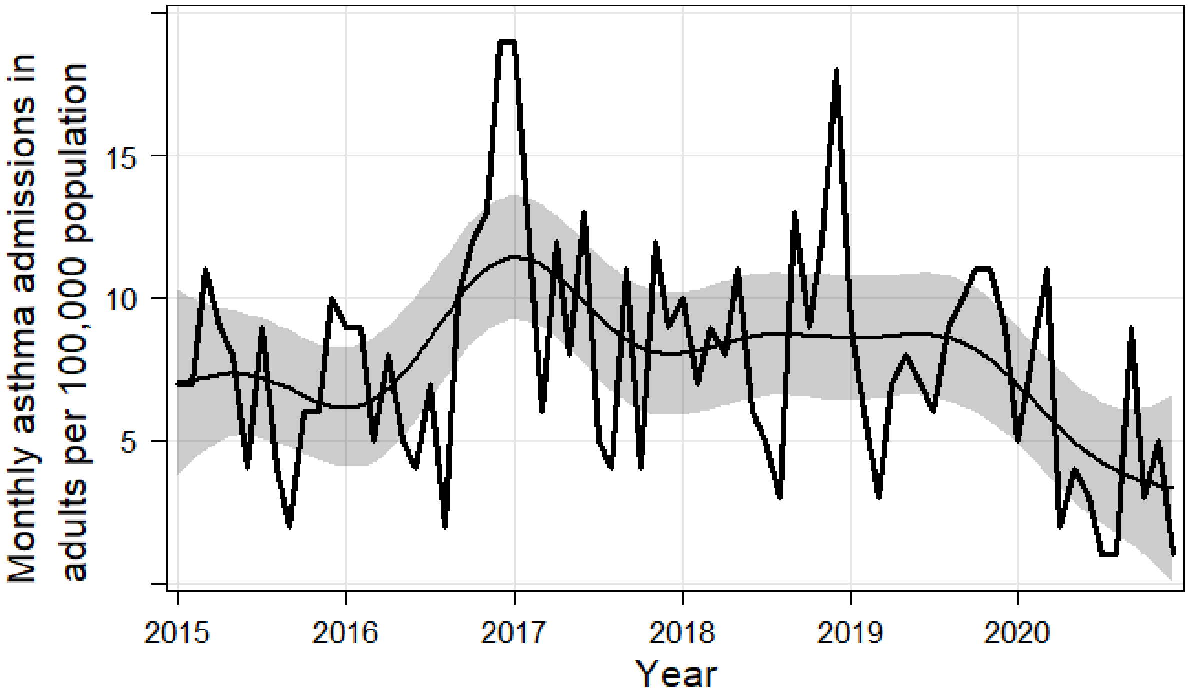

Figure S3 Time series of monthly adult asthma admissions, where the shaded lines represent the smooth fit at the 95% confidence interval.

Table S1 Incidence of adult hospital admissions for acute asthma in 2020 compared with pre-pandemic years (2015 – 2019).

Period	Incidence rate	IRR	95% CI	p value
**Pre-pandemic (2015 – 2019) mean**	78.29	Baseline		
**2020**	45.70	0.58	0.42 – 0.81	<0.001

*per 100,000 population

Table S2 Pearson correlation (r) matrix between air pollutant concentrations & meteorology and adult asthma admissions in 2020 versus 2015 – 2019**.**

Variables	NO_2	$PM_{2.5}$	PM_{10}	Temp	RH
**Asthma admission 2015 – 2019**	0.31	0.27	0.22	0.34	0.30
**Asthma admission 2020**	0.22	0.16	0.20	-0.38	0.14

STROBE Statement—checklist of items that should be included in reports of observational studies

	Item No	Recommendation	
**Title and abstract**	1 ✓	(*a*) Indicate the study's design with a commonly used term in the title or the abstract	(Page 2)
	✓	(*b*) Provide in the abstract an informative and balanced summary of what was done and what was found	(Pages 2, 3)
**Introduction**			
Background/rationale	2 ✓	Explain the scientific background and rationale for the investigation being reported	(Pages 4, 5)
Objectives	3 ✓	State specific objectives, including any prespecified hypotheses	(Page 5)
**Methods**			
Study design	4 ✓	Present key elements of study design early in the paper	(Page 6)
Setting	5 ✓	Describe the setting, locations, and relevant dates, including periods of recruitment, exposure, follow-up, and data collection	(Page 6)
Participants (Page 6)	6 ✓	(*a*) *Cohort study*—Give the eligibility criteria, and the sources and methods of selection of participants. Describe methods of follow-up<br>*Case-control study*—Give the eligibility criteria, and the sources and methods of case ascertainment and control selection. Give the rationale for the choice of cases and controls<br>*Cross-sectional study*—Give the eligibility criteria, and the sources and methods of selection of participants	
		(*b*) *Cohort study*—For matched studies, give matching criteria and number of exposed and unexposed<br>*Case-control study*—For matched studies, give matching criteria and the number of controls per case	
Variables	7 ✓	Clearly define all outcomes, exposures, predictors, potential confounders, and effect modifiers. Give diagnostic criteria, if applicable	(Page 6, 7)
Data sources/ measurement	8* ✓	For each variable of interest, give sources of data and details of methods of assessment (measurement). Describe comparability of assessment methods if there is more than one group	(Pages 6, 7)
Bias	9 ✓	Describe any efforts to address potential sources of bias	(NA)
Study size	10 ✓	Explain how the study size was arrived at	(Page 7)
Quantitative variables	11 ✓	Explain how quantitative variables were handled in the analyses. If applicable, describe which groupings were chosen and why	(Pages 7, 8)
Statistical methods (Pages 7, 8)	12 ✓	(*a*) Describe all statistical methods, including those used to control for confounding	
		(*b*) Describe any methods used to examine subgroups and interactions	
		(*c*) Explain how missing data were addressed	
		(*d*) *Cohort study*—If applicable, explain how loss to follow-up was addressed<br>*Case-control study*—If applicable, explain how matching of cases and controls was addressed<br>*Cross-sectional study*—If applicable, describe analytical methods taking account of sampling strategy	
		(*e*) Describe any sensitivity analyses	

Continued on next page

For peer review only

For peer review only - http://bmjopen.bmj.com/site/about/guidelines.xhtml

**Results**		
Participants (Pages 9,10,11)	13* ✓	(a) Report numbers of individuals at each stage of study—eg numbers potentially eligible, examined for eligibility, confirmed eligible, included in the study, completing follow-up, and analysed
		(b) Give reasons for non-participation at each stage
		(c) Consider use of a flow diagram
Descriptive data (Pages 9-11)	14* ✓	(a) Give characteristics of study participants (eg demographic, clinical, social) and information on exposures and potential confounders
		(b) Indicate number of participants with missing data for each variable of interest
		(c) *Cohort study*—Summarise follow-up time (eg, average and total amount)
Outcome data	15* ✓	*Cohort study*—Report numbers of outcome events or summary measures over time
		Case-control study—Report numbers in each exposure category, or summary measures of exposure
		Cross-sectional study—Report numbers of outcome events or summary measures
Main results (Pages 8-12)	16 ✓	(*a*) Give unadjusted estimates and, if applicable, confounder-adjusted estimates and their precision (eg, 95% confidence interval). Make clear which confounders were adjusted for and why they were included
		(*b*) Report category boundaries when continuous variables were categorized
		(*c*) If relevant, consider translating estimates of relative risk into absolute risk for a meaningful time period
Other analyses	17 ✓	Report other analyses done—eg analyses of subgroups and interactions, and sensitivity analyses (Page 12)
**Discussion**		
Key results	18 ✓	Summarise key results with reference to study objectives (Pages 12-14)
Limitations	19 ✓	Discuss limitations of the study, taking into account sources of potential bias or imprecision. Discuss both direction and magnitude of any potential bias (Page 14)
Interpretation	20 ✓	Give a cautious overall interpretation of results considering objectives, limitations, multiplicity of analyses, results from similar studies, and other relevant evidence (Page 14)
Generalisability	21 ✓	Discuss the generalisability (external validity) of the study results (Pages 12-14)
**Other information**		
Funding	22 ✓	Give the source of funding and the role of the funders for the present study and, if applicable, for the original study on which the present article is based (Page 16)

*Give information separately for cases and controls in case-control studies and, if applicable, for exposed and unexposed groups in cohort and cross-sectional studies.

Note: An Explanation and Elaboration article discusses each checklist item and gives methodological background and published examples of transparent reporting. The STROBE checklist is best used in conjunction with this article (freely available on the Web sites of PLoS Medicine at http://www.plosmedicine.org/, Annals of Internal Medicine at http://www.annals.org/, and Epidemiology at http://www.epidem.com/). Information on the STROBE Initiative is available at www.strobe-statement.org.

For peer review only
